# The Indirect Efficacy Comparison of DNA Methylation in Sputum for Early Screening and Auxiliary Detection of Lung Cancer: A Meta-Analysis

**DOI:** 10.3390/ijerph14070679

**Published:** 2017-06-23

**Authors:** Di Liu, Hongli Peng, Qi Sun, Zhongyao Zhao, Xinwei Yu, Siqi Ge, Hao Wang, Honghong Fang, Qing Gao, Jiaonan Liu, Lijuan Wu, Manshu Song, Youxin Wang

**Affiliations:** 1 Beijing Municipal Key Laboratory of Clinical Epidemiology, School of Public Health, Capital Medical University, Beijing 100069, China; liudiepistat@163.com (D.L.); phongli1990@163.com (H.P.); cmusunqi@163.com (Q.S.); yao_abcde@163.com (Z.Z.); yuxinweigongzuo@163.com (X.Y.); gesky90@sina.com (S.G.); wanghaostudy@163.com (H.W.); fanghonghong79@sina.com (H.F.); shouyigaoqing@163.com (Q.G.); liujiaonan9999@163.com (J.L.); wujuan811017@163.com (L.W.); songms@ccmu.edu.cn (M.S.); 2 School of Medical and Health Sciences, Edith Cowan University, Perth 6027, Australia

**Keywords:** DNA methylation, lung cancer, sputum, detection, meta-analysis, indirect efficacy comparison

## Abstract

Background: DNA methylation in sputum has been an attractive candidate biomarker for the non-invasive screening and detection of lung cancer. Materials and Methods: Databases including PubMed, Ovid, Cochrane library, Web of Science databases, Chinese Biological Medicine (CBM), Chinese National Knowledge Infrastructure (CNKI), Wanfang, Vip Databases and Google Scholar were searched to collect the diagnostic trials on aberrant DNA methylation in the screening and detection of lung cancer published until 1 December 2016. Indirect comparison meta-analysis was used to evaluate the diagnostic value of the included candidate genes. Results: The systematic literature search yielded a total of 33 studies including a total of 4801 subjects (2238 patients with lung cancer and 2563 controls) and covering 32 genes. We identified that methylated genes in sputum samples for the early screening and auxiliary detection of lung cancer yielded an overall sensitivity of 0.46 (0.41–0.50) and specificity of 0.83 (0.80–0.86). Combined indirect comparisons identified the superior gene of *SOX17* (sensitivity: 0.84, specificity: 0.88), *CDO1* (sensitivity: 0.78, specificity: 0.67), *ZFP42* (sensitivity: 0.87, specificity: 0.63) and *TAC1* (sensitivity: 0.86, specificity: 0.75). Conclusions: The present meta-analysis demonstrates that methylated *SOX17*, *CDO1*, *ZFP42*, *TAC1*, *FAM19A4*, *FHIT*, *MGMT*, *p16*, and *RASSF1A* are potential superior biomarkers for the screening and auxiliary detection of lung cancer.

## 1. Introduction

Lung cancer is the leading cause of malignant tumor death, with the morbidity and mortality of lung cancer gradually increasing over the past decades [1]. Only 13% of lung cancer patients survive more than 5 years, and the mortality is close to the morbidity (the ratio of mortality to morbidity is 0.87) [1,2,3]. Despite research on the diagnosis of lung cancer and the use of increasingly advanced technology in its treatment, the prognosis remains poor because of the predominant diagnosis of III, IV-stage disease [3]. Therefore, early diagnosis, auxiliary detection, and treatment have become a major focus to reduce the mortality caused by lung cancer.

With the emergence and development of molecular epidemiology, which opens the “black box” of the disease process, molecular biomarkers have much potential to improve the understanding of the occurrence, development, and prognosis of disease for the early detection of these lesions at the pre-invasive stage, even predicting the progress of the disease [4,5]. Nowadays, it is clearly acknowledged that genetic alterations are accompanied by equally important epigenetic modifications in the pathogenesis of lung cancer [6,7]. In particular, DNA methylation is one of the earliest epigenetic modifications; it is closely associated with the occurrence and development of lung cancer, and it appears earlier than obvious malignant phenotype [7]. Abundant evidence manifests that a variety of well-known DNA methylations can be used as a promising biomarker for the early diagnosis of lung cancer, such as *p16* [8], *RASSF1A* [9], *APC* [10], *MGMT* [11], *DAPK* [12] and *RARβ* [13]. Many studies have demonstrated that the aberrant methylation of some genes in sputum samples could be a novel “remote medium” for the early detection of lung cancer, which avoids the necessity of invasive procedures [5,14]. The development of next-generation sequencing technology and the maturation of methylation detection technology [15,16] have made methylation detection much more stable and cheaper, and could provide the necessary conditions for clinical utility. Taken together, findings suggest that methylation could serve as efficient diagnostic biomarkers for lung cancer.

However, the DNA methylation of specific genes in sputum samples do not yet provide a convincing and superior gene panel with high sensitivity and specificity for clinical utility in lung cancer. In addition, there was still no comparative evaluation on the diagnostic accuracy of methylation in sputum samples for the screening and detection of lung cancer. Recently, network meta-analysis has been developed to assess the comparative effectiveness of several interventions and to synthesize evidence across a network of studies [17,18]. This method makes it possible to estimate the comparative diagnostic accuracy of multiple biomarkers which have not been directly compared with each other in one study but have been reported by multiple studies under a common comparator. Therefore, we performed a network meta-analysis to indirectly compare the efficacy of the DNA methylation of multiple genes in the diagnosis of lung cancer.

## 2. Materials and Methods

We followed the reporting guidelines of the Preferred Reporting Items for Systematic Reviews and Meta-Analysis (PRISMA) statement when conducting this review. The PRISMA statement has guidelines that include a four-phase flow diagram to systematically guide the inclusion and exclusion of research papers [19]. In addition, the guidelines provide a 27-item checklist that describes the requirements per review section (e.g., title, abstract, introduction, methods, results, discussions, and funding) to ensure that systematic reviews are properly conducted and reported [19].

### 2.1. Search Strategy

We conducted a comprehensive literature search in PubMed, Ovid, Cochrane library, Web of Science databases, Chinese Biological Medicine (CBM), Chinese National Knowledge Infrastructure (CNKI), Wanfang, Vip Databases, and Google Scholar. The main search terms included: lung cancer or lung carcinoma or non-small cell lung cancer or “NSCLC”, “sputum or flema”, “diagnostic”, “sensitivity and specificity” and “methylation or hypermethylation or hypomethylation or demethylation”. All articles published until December 2016 were considered. In addition, the reference lists of all identified studies were manually searched to identify any additional studies.

### 2.2. Inclusion Criteria and Exclusion Criteria

The articles, which could not be excluded based on the title and abstract, were retrieved for full-text review. Studies were included in this meta-analysis if they met the following criteria: (1) the diagnostic potential of sputum DNA methylation for lung cancer; (2) study design being case-control; (3) the patients being diagnosed with lung cancer by pathology; (4) provided data on the numbers of true positives (TP), false positives (FP), true negatives (TN), and false negatives (FN); (5) the methods of detecting methylation based on methylation-specific polymerase chain reaction (MSP) or quantitative methylation-specific PCR (qMSP).

Studies were excluded from the meta-analysis for the following reasons: (1) abstracts, letters, reviews, expert opinions, case reports, or nonclinical studies; (2) studies had duplicate or overlapping data; (3) study was based on tissue, blood, or animals.

### 2.3. Data Extraction and Quality Assessment

Data extraction was conducted in duplicate by two investigators (Di Liu and Hongli Peng) based on title, abstract, author, year of publication, country of origin, sample size, assay methods, and diagnostic performance (sensitivity (SEN), specificity (SPE), TP, FP, FN, TN), target gene(s), and the score of the quality assessment of studies of diagnostic accuracy (QUADAS) [20] and the standards for reporting of diagnostic accuracy (STARD) [21]. Any disagreements in data extraction were resolved by consensus.

STARD and QUADAS guidelines were utilized to assess the methodological quality of each study. There are 25 items in the STARD initiative checklist, and a score of 1 was given when the item was yielded [21]. Fourteen items were included in the QUADAS tool, whereby a score of 1 was given when a specific item was fulfilled, 0 if this item was unclear, and −1 for the item not achieved [20]. All of these studies were evaluated independently and discussed by the reviewers until a consensus was reached. 

### 2.4. Statistical Analysis

We used standard methods recommended for the direct meta-analysis which estimated the diagnostic test evaluation of DNA methylation compared with the gold standard [22]. The number of TP, TN, FP, and FN were retrieved from each article. The SEN, SPE, positive likelihood ratio (PLR), negative likelihood ratio (NLR), diagnostic odds ratio (DOR) estimates with 95% confidence interval (CI) from each study were analyzed using a random-effect model and the bivariate summary receiver operating characteristic (SROC) curve was generated. The area under the curve (AUC) represents an analytical summary of the test performance and illustrates the trade-off between sensitivity and specificity [23]. The heterogeneity among studies was assessed on the basis of the Chi Square test using the Cochran Q statistic. The *I*^2^ statistic, which measures the extent of inconsistency between studies, was also assessed [24]. Spearman’s correlation coefficient of logarithm sensitivity and 1-specificity for each gene was assessed to determine the threshold effect [25]. Analyses were performed using two statistical software programs (Meta-Disc 1.4 for Windows and Stata version 12.0, Stata Corp, College Station, TX, USA).

For indirect comparisons, the comparative diagnostic accuracy of all biomarkers was estimated according to common comparator (the gold standard). We did not assume consistency (which was evaluated by comparing the direct estimates with the indirect estimates for each comparison) of two biomarkers without direct analyses. We took the step-wise approach, which was suitable for the simple star-network meta-analysis to obtain an indirect analysis. The Deeks’ test and Egger’s test were utilized to estimate the funnel plot asymmetry and the publication bias [26]. The indirect meta-analysis was conducted using indirect treatment comparison (ITC) software and Stata 12.0 (Stata Corp, College Station, TX, USA) [27]. A two-sided *p*-value of less than 0.05 was considered significant.

## 3. Results

### 3.1. Subsection

#### 3.1.1. Study Characteristics and Quality of Included Studies

**Figure 1:**
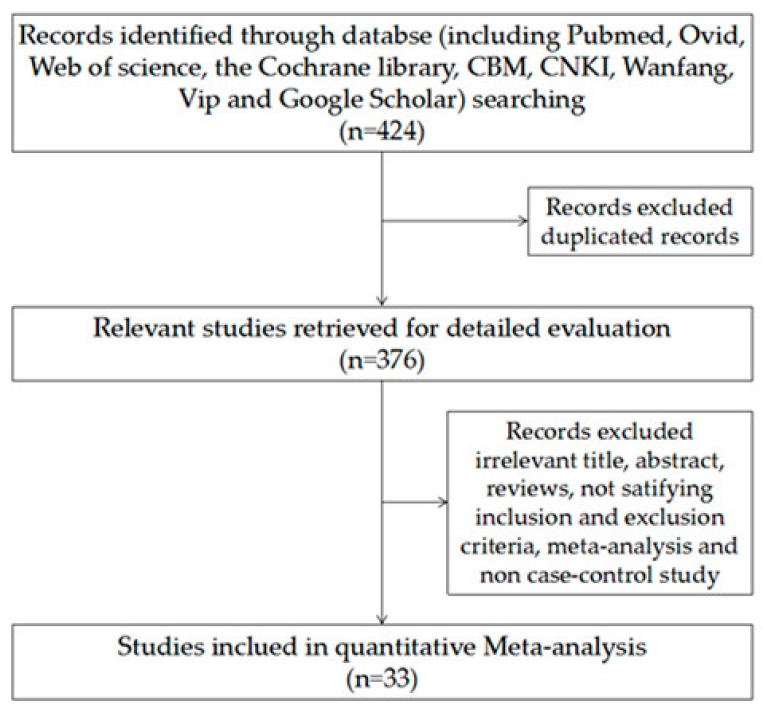
Flow diagram of studies through the review process.

The flowchart of included studies was presented in Figure 1. A total of 424 studies were preliminarily reviewed, of which 33 were available for the indirect meta-analysis [5,14,28,29,30,31,32,33,34,35,36,37,38,39,40,41,42,43,44,45,46,47,48,49,50,51,52,53,54,55,56,57,58]. The characteristics of each study are shown in Table 1, including name of the study, number of patients and controls, biomarkers, and quality assessment based on STARD and QUADAS. The systematic literature search yielded a total of 33 studies including a total of 4801 subjects (2238 patients with lung cancer and 2563 controls) and covering 32 genes (*P16*, *RASSF1A*, *APC*, *MGMT*, *PAX5*, *CGB*, *GATA*, *DAPK*, *RARβ*, *MAGE*, *HOXA*, *3OST2*, *PRDM14*, *FAM19A4*, *PHACTR3*, *PCDH20*, *CXCL*, *Dal-1*, *Dab2*, *Dcr2*, *SULF2*, *Kifla*, *Jph3*, *SOX17*, *CDO1*, *ZFP42*, *TAC1*, *CDH1*, *H-cadherin*, *FHIT*, *RASSF2*, *TCF2l*).

#### 3.1.2. Summary Performance of Diagnostic Estimates

As shown in Table 2, of the 32 genes, the SEN (sensitivity) ranged from 0.03 to 0.87 (pooled 0.46; 95% CI: 0.41–0.50), whereas SPE ranged from 0.25 to 0.99 (pooled 0.83; 95% CI: 0.80–0.86).The PLR (positive likelihood ratio) ranged from 1.03 to 6.76 (pooled 2.72; 95% CI: 2.32–3.22),NLR (negative likelihood ratio) ranged from 0.18 to 0.98 (pooled 0.64; 95% CI: 0.60–0.68), and DOR (diagnostic odds ratio) ranged from 1.05 to 38.00 (pooled 4.28; 95% CI: 3.50–5.20). Of the 32 genes, the higher of SEN were *ZFP42*: 0.87 (0.78–0.93), *TAC1*: 0.86 (0.77–0.92), *SOX17*: 0.84 (0.75–0.91), *FAM19A4*: 0.80 (0.74–0.85), and *HOXA*: 0.79 (0.63–0.89); whereas the higher of PLR were *SOX17*: 6.76 (2.34–19.54), *FHIT*: 5.93 (2.29–15.36), *RASSF1A*: 5.61 (3.73–8.43), *MGMT*: 4.78 (1.47–15.55), and *p16*: 4.71 (2.53–8.78).

**Figure 2:**
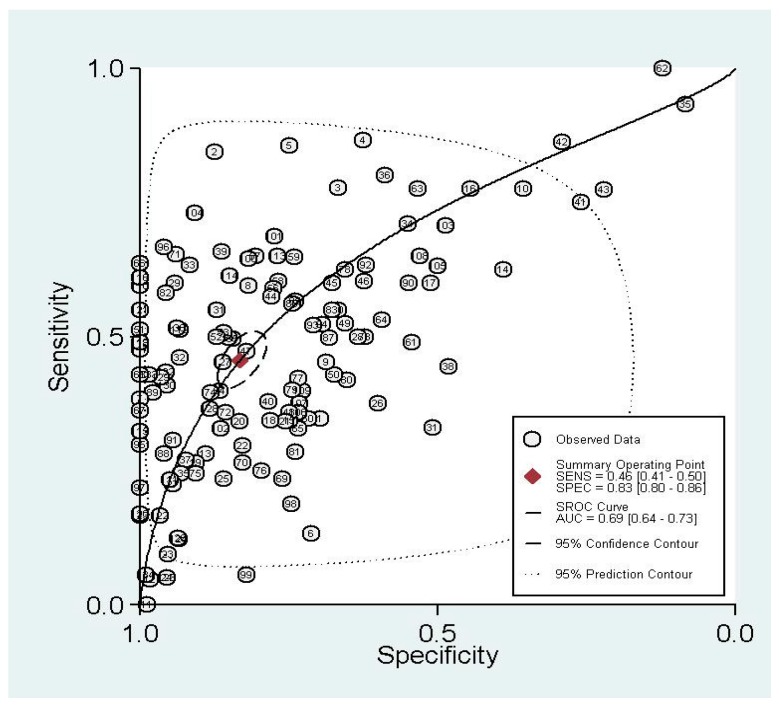
Summary receiver operating characteristic (SROC) curve with pooled estimates of sensitivity, specificity, and area under the curve (AUC) on the diagnostic value of gene methylation in lung cancer.

The graph of the SROC (summary receiver operating characteristic) cure is shown in Figure 2, which demonstrates the trade-off between SEN and SPE values in multiple studies. The SROC curve results showed that AUC (area under the curve) of 32 different methylated genes was 0.69 (0.64–0.73), indicating the ability of 32 pooled gene methylations to differentiate lung cancer patients from non-lung-cancer patients with a mid-level accuracy.

#### 3.1.3. Indirect Comparisons of Diagnostic Analysis

**Figure 3:**
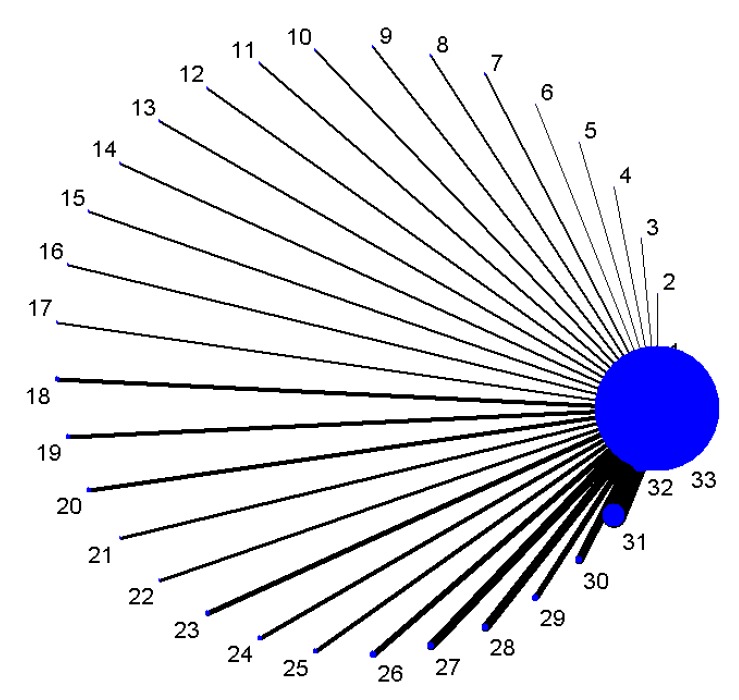
The star-network plot of different methylated genes for 1–32 (1/*CDH1*, 2/*SOX17*, 3/*CDO1*, 4/*ZFP42*, 5/*TAC1*, 6/*H-cadherin*, 7/*FHIT*, 8/*PCDH20*, 9/*Dab2*, 10/*Dcr2*, 11/*SULF2*, 12/*Kifla*, 13/*Dal-1*, 14/*Jph3*, 15/*RASSF2*, 16/*TCF2l*, 17/*CXCL*, 18/*MAGE*, 19/*HOXA*, 20/*RARβ*, 21/*FAM19A4*, 22/*PHACTR3*, 23/*DAPK*, 24/*3OST2*, 25/*PRDM14*, 26/*GATA*, 27/*MGMT*, 28/*PAX5*, 29/*CYGB*, 30/*APC*, 31/*p16*, 32/*RASSF1A*) and 33/the gold standard of being diagnosed with lung cancer.

Figure 3 shows the star-network of comparisons for methylated genes (1–32) with the gold standard of being diagnosed with lung cancer (33). We established a network to compare the diagnostic accuracy of 32 methylated genes, with the results of indirect comparisons presented in Table S1. The OR and 95% CI of *SOX17*, *TAC1*, *ZFP42*, *CDO1* differed significantly from the other 28 methylated genes and were higher than 1, indicating that *SOX17*, *TAC1*, *ZFP42*, and *CDO1* have a higher diagnostic accuracy. More information is shown in the supplemental content (Figures S1–S32). We combined indirect comparisons to evaluate the comparative efficacy of different methylated genes; the superior genes were performed by *SOX17* (sensitivity: 0.84, specificity: 0.88), *CDO1* (sensitivity: 0.78, specificity: 0.67), *ZFP42* (sensitivity: 0.87, specificity: 0.63), *TAC1* (sensitivity: 0.86, specificity: 0.75) (Table 2).

#### 3.1.4. Test of Heterogeneity and Meta-Regression

In the meta-analysis, computation of the Spearman’s rank correlation coefficient between the logit of sensitivity and that of 1-specificity of sputum DNA testing was 0.465 (*p* < 0.001), indicating the heterogeneity of threshold effect. We also investigated the non-threshold effects; the results indicated the existence of significant heterogeneity in the overall sensitivity (*I*^2^ = 91.2%, *p* < 0.001), specificity (*I*^2^ = 93.5%, *p* < 0.001), PLR (*I*^2^ = 85.4%, *p* < 0.001), NLR (*I*^2^ = 88.8%, *p* < 0.001), and DOR (*I*^2^ = 72.6%, *p* < 0.001). Therefore, a bivariate binomial mixed model was applied to summarize the pooled estimates in this study. To determine the sources of heterogeneity, we performed meta-regression to test the effect of ethnicity (Asian/others), sample size (*n* = 0–100/101–200/201–), and the quality of study (low/medium/high). Multivariable regression showed that ethnicity (coefficient = −0.785, *p* = 0.001) and the sample size (coefficient = −0.324, *p* = 0.036) had statistically significant differences, while the quality of study (coefficient = −0.074, *p* = 0.552) showed no significant difference. Then, we conducted subgroup analysis based on ethnicity and the sample size, as shown in Table 3.

In addition, we performed a meta-regression to test the effect of ethnicity, sample size, and the quality of study with different genes. The results showed that ethnicity (coefficient = −1.117, *p* = 0.048) and the sample size (coefficient = −1.177, *p* = 0.026) were of statistically significant bias for *p16*, while not significant bias for other candidate genes (*p* > 0.050).

**Figure 4:**
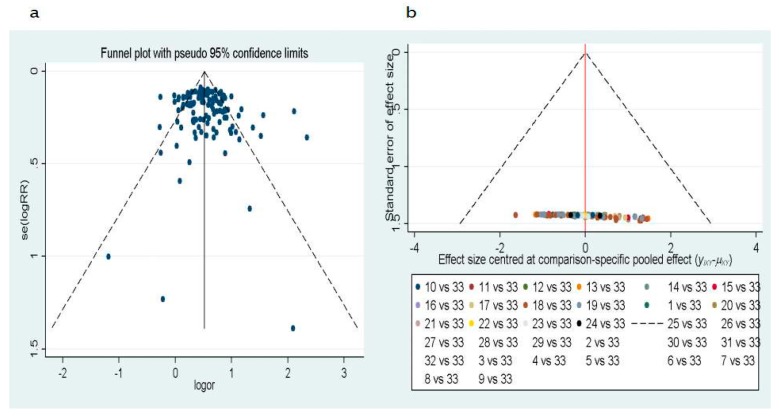
Funnel plot of the publication bias (**a**) from Deeks’ test, the data is based on the article of cases and controls. (**b**) From Egger’s test, the data is based on the article indirect comparisons of methylated gene for 1–32 (1/*CDH1*, 2/*SOX17*, 3/*CDO1*, 4/*ZFP42*, 5/*TAC1*, 6/*H-cadherin*, 7/*FHIT*, 8/*PCDH20*, 9/*Dab2*, 10/*Dcr2*, 11/*SULF2*, 12/*Kifla*, 13/*Dal-1*, 14/*Jph3*, 15/*RASSF2*, 16/*TCF2l*, 17/*CXCL*, 18/*MAGE*, 19/*HOXA*, 20/*RARβ*, 21/*FAM19A4*, 22/*PHACTR3*, 23/*DAPK*, 24/*3OST2*, 25/*PRDM14*, 26/*GATA*, 27/*MGMT*, 28/*PAX5*, 29/*CYGB*, 30/*APC*, 31/*p16*, 32/*RASSF1A*) and 33/the gold standard of being diagnosed with lung cancer.

Publication bias was evaluated by Deeks’ test and Egger’s test. The funnel plots for publication bias showed no asymmetry (Figure 4). The result of Deeks’ test showed that *p* = 0.008, indicating that publication bias could exist in the meta-analysis.

## 4. Discussion

Lung cancer has become a global burden, further substantiating the need for early screening and auxiliary detection [1,3]. The key to accomplishing both these goals is the better understanding of the genes or pathways disrupted in causing lung cancer [6,45,59]. The fact that silencing genes through hypermethylation or activating genes through hypomethylation play an important role in the initiation and progression of lung cancer has stimulated the development of screening approaches to identify additional genes and pathways that are disrupted within the epigenome [59]. In addition, DNA methylation in sputum samples has the potential to serve as a non-invasive screening method for the identification of specific biomarkers, enabling the early detection of lung cancer [31,39].

In the direct meta-analysis, we identified that methylated genes in sputum samples for the early screening and auxiliary detection of lung cancer yielded an overall sensitivity of 0.46 at the same specificity of 0.83. Furthermore, the PLR (positive likelihood ratio) was 2.72, NLR (negative likelihood ratio) was 0.64, and DOR (diagnostic odds ratio) value was 4.28, and the AUC (area under the curve) was 0.69, indicating a mid-level accuracy. Therefore, we should pick the superior genes for clinical utility as diagnostic biomarkers for lung cancer. Combined indirect comparisons identified the superior genes as *SOX17* (sensitivity: 0.84, specificity: 0.88), *CDO1* (sensitivity: 0.78, specificity: 0.67), *ZFP42* (sensitivity: 0.87, specificity: 0.63), and *TAC1* (sensitivity: 0.86, specificity: 0.75). A single DNA methylation biomarker cannot be expected to detect all cases of lung cancer. Some studies demonstrated that combined multiple methylated genes could improve the diagnostic value of cancers [37,60]. We identified that the sensitivity value of methylated *FAM19A4* and PLR value of methylated *RASSF1A*, *FHIT*, *MGMT*, and *p16* are relatively high, suggesting that they are comprehensive parameters for the screening test [61]. In addition, methylated *RASSF1A* and *p16* genes are reported to be promising driving molecules in many cancers under the concept of precision medicine [9,62,63,64,65,66,67]. In addition, the methylation of *FAM19A4*, *FHIT*, and *MGMT* were reported to play important roles in the occurrence and deterioration of lung cancer [68,69,70,71]. Therefore, we advocate that methylated *SOX17*, *CDO1*, *ZFP42*, *TAC1*, *FAM19A4*, *FHIT*, *MGMT*, *p16*, and *RASSF1A* are useful in the screening and auxiliary detection of lung cancer.

To our knowledge, this study is the first systemic review and indirect meta-analysis to assess the comparative diagnostic effectiveness of the methylation profile of multiple candidate gens in sputum samples for the early screening and detection of lung cancer. According to the method of network meta-analysis [18,27], we used indirect comparison to estimate the comparative diagnostic accuracy of two methylated genes based on a common comparator (the gold standard of being diagnosed with lung cancer). Therefore, the inconsistency between the direct and indirect comparison is not available to address.

However, we observed a large degree of heterogeneity among studies investigating methylation profile in sputum samples used for lung cancer. Threshold effect is one of the primary causes of heterogeneity among diagnostic accuracy studies [24]. In the present meta-analysis, we found obvious heterogeneity as a result of threshold effect, which may be caused by different genes. There is a clear and unified cut-off value for methylation/unmethylation regardless of whether it is based on qualitative analysis or quantitative analysis for each gene tested by the method of MSP [16]. Moreover, we performed meta-regression to test the heterogeneity caused by ethnicity, sample size, and the quality of study. The results suggested that study region (*p* = 0.001) and the sample size (*p* = 0.036) might be a source of heterogeneity for this meta-analysis. The results of subgroup analyses showed that large-sample studies had higher sensitivity than the small- and moderate-sample studies, while studies in Asia had lower sensitivity than other regions. 

In addition, publication bias could exist in the meta-analysis. This meta-analysis was only based on published studies, therefore inducing the possibility of publication bias. The Deeks’ test and Egger’s test not only detect publication bias, but also indicate the heterogeneity due to the effect of ethnicity, sample size, the quality of study, etc. [72,73]. Therefore, we proposed that the heterogeneity was potentially due to different genes, study region, and sample size. However, this speculation needs to be investigated in the future study.

The present network meta-analysis included 33 articles and 32 candidate genes, with the majority of genes only included in one article. These make it difficult to directly compare the diagnostic efficacy among multiple genes; thus, only indirect comparisons were evaluated in this study. However, the absence of direct comparisons may lead to bias. Pairwise meta-analysis and network meta-analysis were carried out sequentially for direct and indirect comparisons of migraine headache days among three interventions compared with those treated by three placebos, and the results showed that there was no significant inconsistency between the direct and indirect evidence for the majority of comparisons [74]. Another network meta-analysis was performed to directly and indirectly compare the effectiveness of several oral antidiabetic drugs in the prevention of cardiovascular mortality and morbidity, and the results indicated that the inconsistency between direct and indirect estimates of all-cause mortality, cardiovascular-related mortality, acute coronary syndrome, and myocardial infraction were significant low [75]. In summary, the present results from indirect comparisons should be reliable and acceptable.

Based on the focus of diagnostic accuracy studies, we identified other common limitations and insufficiency. Firstly, all the publications included in this analysis were reported on case-control studies, indicating that the selection bias could possibly lead to over-estimations of diagnostic accuracy compared with the cross-sectional study and cohort study [76,77]. In addition, the effects of language selection bias and publication bias cannot be ignored in any meta-analysis [19]. Finally, the detection utilizing sputum DNA testing was not good enough. We think two methods might have the potential to screen valid and good biomarkers for the advancement of the field. Firstly, a panel with multiple methylated genes may be of high performance in diagnostic models. Secondly, instead of qualitative methods (MSP), quantitative methods for the determination of the methylation patterns in candidate genes may increase the diagnostic performance.

## 5. Conclusions

In conclusion, despite these limitations, our meta-analysis advocates that methylated *SOX17*, *CDO1*, *ZFP42*, *TAC1*, *FAM19A4*, *FHIT*, *MGMT*, *p16*, and *RASSF1A* are useful biomarkers in the screening and auxiliary detection of lung cancer. Our findings provide new avenues for assessing the comparative diagnostic effectiveness of several methylations in lung cancer based on the method of network meta-analysis. Further high-quality and large-scale studies are needed to confirm our analysis.

## Figures and Tables

**Table 1:** Baseline characteristics of all included studies.

Study/Year	Country	Cases/Controls	Biomarkers	STARD	QUADAS
Destro/2004 [41]	Italy	24/100	*p16*	21	12
Zhang/2004 [48]	China	44/20	*p16*	18	8
Wang/2004 [57]	China	34/21	*p16*	17	7
Konno/2004 [31]	Japan	78/94	*p16*, *APC*, *RARβ*	20	11
Belinsky/2005 [39]	USA	53/118	*p16*, *MGMT*, *RASSF1A*, *DAP*, *H-cadherin*, *PAX5*	20	9
Olaussen/2005 [32]	France	20/17	*HOXA9*, *p16*, *MAGE*	17	8
Cirincione/2006 [44]	Italy	18/112	*RARβ*, *p16*, *RASSF1A*	15	7
Wang/2006 [45]	China	79/22	*FHIT*, *p16*, *RARβ*	20	10
Liu/2006 [54]	China	77/30	*MGMT*	18	8
Belinsky/2006 [47]	USA	98/92	*p16*, *PAX5*, *MGMT*, *DAPK*, *GATA*, *RASSF1ASFRP1*, *HLHPBETA3*, *IGFBP3HCAD*, *LAMC2*	20	10
Georgiou/2007 [30]	Greece	80/40	*p16*	16	9
Hsu/2007 [5]	China	82/37	*p16*, *RARβ*	16	7
Liu/2008 [29]	China	58/107	*p16*	18	10
Guo/2008 [53]	China	100/50	*p16*	16	7
Van der Drift/2008 [33]	Netherlands	28/68	*RASSF1A*	19	11
Hu/2009 [51]	China	42/25	*p16*	16	9
Ye/2010 [49]	China	30/27	*RASSF1A*	16	7
Zhang/2010 [49]	China	82/25	*RASSF1A*, *p16*, *DAPK*	18	8
Hwang/2011 [36]	Korea	76/109	*HOXA*	20	9
Song/2011 [28]	China	42/9	*p16*, *MGMT*	17	8
Zhang/2011 [52]	China	41/15	*p16*	17	9
Hang/2011 [38]	China	47/24	*FHIT*	20	8
Sun/2012 [55]	China	120/120	*p16*, *RASSF1A*	19	7
Hubers/2012 [46]	The Netherlands	53/47	*RASSF1A*, *APC*, *CYGB*	18	10
Guzmán/2012 [14]	Chile	26/33	*p16*, *CDH1*, *MGMT*	18	11
Shin/2012 [42]	Korea	65/30	*MAGE*, *p16*	17	9
Leng/2012 [35]	USA	64/64	*p16*, *MGMT*, *DAPK*, *PAX5*, *GATA*, *Dal-1*, *PCDH20*, *Jph3*, *Kifla*, *SULF2*, *RASSFlA*, *GATA*, *Dab2*, *Dcr2*, *RASSF2*, *TCF2l*	20	11
Leng/2012 [35]	USA	40/90	*p16*, *MGMT*, *DAPK*, *PAX5*, *GATA*, *Dal-1*, *PCDH20*, *Jph3*, *Kifla*, *SULF2*, *CXCL*, *RASSFlA*, *Dab2*, *Dcr2*, *RASSF2*, *TCF2l*	20	11
Pan/2013 [56]	China	20/13	*p16*	19	8
Hubers/2014 [34]	The Netherlands	20/31	*RASSF1A*, *APC*, *CYGB*, *3OST*, *PRDM14*, *FAM19A4*, *PHACTR3*	19	8
Hubers/2014 [43]	The Netherlands	98/90	*RASSF1A*, *APC*, *CYGB*	20	10
Hubers/2014 [43]	The Netherlands	60/445	*RASSF1A*, *APC*, *CYGB*	20	10
Hubers/2015 [58]	The Netherlands	73/86	*RASSF1A*, *APC*, *CYGB*, *3OST2*, *PRDM14*, *FAM19A4*, *PHACTR3*	21	11
Hubers/2015 [58]	The Netherlands	159/154	*RASSF1A*, *APC*, *CYGB*, *3OST2*, *PRDM14*, *FAM19A4*, *PHACTR3*	21	11
Su/2016 [40]	China	117/174	*RASSF1A*, *3OST2*, *PRDM14*	18	7
Hulbert/2016 [37]	USA	90/24	*SOX17*, *TAC1*, *CDO1*, *HOXA*, *ZFP42*	16	9

STARD: Standards for reporting of diagnostic accuracy; QUADAS: Quality assessment of studies of diagnostic accuracy

**Table 2:** The summary performance of diagnostic estimates.

Genes	Study-Case/Control	SEN (95% CI)	SPE (95% CI)	PLR (95% CI)	NLR (95% CI)	DOR (95% CI)
*CDH1*	1–26/33	0.35 (0.17–0.56)	0.70 (0.51–0.84)	1.14 (0.55–2.39)	0.94 (0.66–1.00)	1.22 (0.41–3.65)
*SOX17*	1–90/24	**0.84 (0.75–0.91**	0.88 (0.68–0.97)	**6.76 (2.34–19.54)**	0.18 (0.11–0.29)	38.00 (9.98–144.73)
*CDO1*	1–90/24	0.78 (0.68–0.86)	0.67 (0.45–0.84)	2.32 (1.31–4.15)	0.33 (0.21–0.54)	7.00 (2.62–18.72)
*ZFP42*	1–90/24	**0.87 (0.78–0.93)**	0.63 (0.41–0.81)	2.31 (1.37–3.90)	0.21 (0.12–0.39)	10.83 (3.88–30.22
*TAC1*	1–90/24	**0.86 (0.77–0.92)**	0.75 (0.53–0.90)	3.42 (1.70–6.88)	0.19 (0.11–0.33)	17.77 (5.94–53.12)
*H-cadherin*	1–53/118	0.50 (0.23–0.77)	0.57 (0.46–0.68)	1.18 (0.66–2.11)	0.87 (0.50–1.00)	1.35 (0.43–4.22)
*FHIT*	2–126/46	0.52 (0.43–0.61)	0.91 (0.79–0.98)	**5.93 (2.29–15.36)**	0.53 (0.43–0.65)	11.19 (3.79–33.06)
*PCDH20*	2–104/154	0.58 (0.48–0.67)	0.49 (0.41–0.58)	1.14 (0.91–1.43)	0.86 (0.65–1.00)	1.33 (0.80–2.19)
*Dab2*	2–104/154	0.03 (0.01–0.08)	0.99 (0.95–1.00)	2.22 (0.38–13.06)	0.98 (0.95–1.00)	2.26 (0.37–13.75)
*Dcr2*	2–104/154	0.41 (0.32–0.51)	0.60 (0.52–0.68)	1.03 (0.76–1.38)	0.98 (0.80–1.00)	1.05 (0.63–1.73)
*SULF2*	2–104/154	0.51 (0.41–0.61)	0.57 (0.49–0.65)	1.19 (0.91–1.55)	0.86 (0.68–1.00)	1.39 (0.84–2.28)
*Kifla*	2–104/154	0.44 (0.34–0.54	0.62 (0.54–0.70	1.17 (0.87–1.58)	0.89 (0.72–1.00)	1.31 (0.79–2.18)
*Dal-1*	2–104/154	0.30 (0.21–0.39	0.86 (0.80–0.91	2.17 (1.32–3.55)	0.82 (0.71–0.94)	2.65 (1.42–4.94)
*Jph3*	2–104/154	0.31 (0.23–0.41	0.79 (0.721–0.85	1.47 (0.97–2.22)	0.87 (0.75–1.00)	1.68 (0.96–2.95)
*RASSF2*	2–104/154	0.08 (0.03–0.15)	0.95 (0.91–0.98)	1.69 (0.63–4.52)	0.97 (0.91–1.00)	1.75 (0.61–4.98)
*TCF2l*	2–104/154	0.29 (0.20–0.39)	0.71 (0.63–0.78)	0.99 (0.67–1.46)	1.01 (0.86–1.00)	0.98 (0.57–1.70)
*CXCL*	2–80/180	0.36 (0.26–0.48	0.79 (0.72–0.85	1.72 (1.15–2.58)	0.81 (0.67–0.97)	2.12 (1.19–23.79)
*MAGE*	4–202/118	0.45 (0.34–0.55	0.82 (0.56–0.94	2.44 (0.75–7.96	0.68 (0.47–0.99	3.60 (0.76–16.98
*HOXA*	4–276/174	**0.79 (0.63–0.89)**	0.50 (0.16–0.84)	1.56 (0.80–3.07)	0.43 (0.28–0.66)	3.63 (1.28–10.26)
**RARβ**	4–257/223	0.44 (0.29–0.60)	0.79 (0.58–0.91)	2.09 (0.93–4.70)	0.71 (0.52–0.98)	2.93 (0.99–8.69)
*FAM19A4*	3–252/271	**0.80 (0.74–0.85**	0.25 (0.20–0.30)	1.06 (0.97–1.16)	0.82 (0.59–1.00)	1.29 (0.86–1.96)
*PHACTR3*	3–252/271	0.60 (0.53–0.66	0.68 (0.62–0.73)	1.85 (1.52–2.27)	0.60 (0.50–0.71)	3.11 (2.17–4.45)
*DAPK*	5–337/389	0.45 (0.40–0.51)	0.79 (0.64–0.89)	2.16 (1.13–4.14)	0.69 (0.58–0.86)	3.12 (1.32–7.36)
*3OST2*	4–369/445	0.50 (0.45–0.55)	0.85 (0.82–0.88)	3.36 (2.63–4.30)	0.59 (0.53–0.66)	5.71 (4.10–7.96)
*PRDM14*	4–369/445	0.62 (0.57–0.67)	0.76 (0.72–0.80)	2.63 (2.19–3.17)	0.50 (0.43–0.57)	5.30 (3.91–7.17)
*GATA*	6–404/492	0.66 (0.31–0.90)	0.53 (0.33–0.71)	1.40 (1.16–1.69)	0.64 (0.34–1.21)	2.20 (1.01–4.83)
*MGMT*	8–447/460	0.42 (0.32–0.52)	0.91 (0.77–0.97)	**4.78 (1.47–15.55)**	0.64 (0.50–0.81)	7.48 (1.87–29.91)
*PAX5*	8–510/728	0.37 (0.29–0.45)	0.78 (0.70–0.84)	1.65 (1.28–2.12)	0.81 (0.74–0.90)	2.02 (1.45–2.83)
*CYGB*	6–453/853	0.51 (0.45–0.57)	0.79 (0.69–0.88)	2.39 (1.61–3.56)	0.62 (0.54–0.72)	3.83 (2.28–6.44)
*APC*	8–588/928	0.43 (0.34–0.53)	0.87 (0.71–0.95)	3.30 (1.67–6.51)	0.65 (0.59–0.72)	5.06 (2.55–10.04)
*p16*	24–1357/1249	0.48 (0.40–0.56)	0.90 (0.82–0.95)	**4.71 (2.53–8.78)**	0.58 (0.50–0.68)	8.11 (3.94–16.70)
*RASSF1A*	17–1160/1767	0.28 (0.20–0.38)	0.95 (0.93–0.97)	**5.61 (3.73–8.43)**	0.76 (0.67–0.85)	7.40 (4.54–12.06)
Summary	33–2238/2563	0.46 (0.41–0.50)	0.83 (0.80–0.86)	2.72 (2.32–3.22)	0.64 (0.60–0.68)	4.28 (3.50–5.20)

DOR: Diagnostic odds ratio; NLR: Negative likelihood ratio; PLR: Positive likelihood ratio; SEN: Sensitivity; SPE: Specificity. Bold text: The top five values.

**Table 3:** Detailed information of subgroup analysis.

Analysis	SEN (95% CI)	SPE (95% CI)	PLR (95% CI)	NLR (95% CI)	DOR (95% CI)
Ethnicity					
Asian	0.46 (0.44–0.48)	0.84 (0.93–0.86)	4.04 (2.91–5.62)	0.61 (0.55–0.66)	6.50 (4.73–8.92)
Others	0.47 (0.46–0.48)	0.75 (0.74–0.75)	1.88 (1.68–2.10)	0.72 (0.68–0.77)	2.92 (2.46–3.47)
Sample size					
0–100	0.48 (0.46–0.51)	0.85 (0.83–0.88)	4.13 (2.80–6.08)	0.59 (0.54–0.65)	7.80 (5.22–11.65)
101–200	0.45 (0.44–0.46)	0.75 (0.74–0.76)	1.78 (1.58–2.00)	0.73 (0.69–0.78)	2.75 (2.29–3.31)
201–	0.51 (0.48–0.53)	0.76 (0.74–0.77)	2.52 (1.85–3.44)	0.65 (0.59–0.73)	3.94 (2.68–5.42)

## Supplementary Materials

The following are available online at www.mdpi.com/1660-4601/14/7/679/s1, Figures S1–S32: Indirect comparisons between one methylated gene and 31 other genes; Table S1: Indirect comparisons of 32 genes.
