# Supplementary material for: The Indirect Efficacy Comparison of DNA Methylation in Sputum for Early Screening and Auxiliary Detection of Lung Cancer: A Meta-Analysis
Source: Int J Environ Res Public Health. 2017 Jun 23;14(7):679. doi: 10.3390/ijerph14070679 (PMC5551117; doi:10.3390/ijerph14070679)
Supplement: Supplementary file 1 [file ijerph-14-00679-s001.pdf]

Supplementary Figures: Indirect comparisons between one methylated gene and 31 other genes.

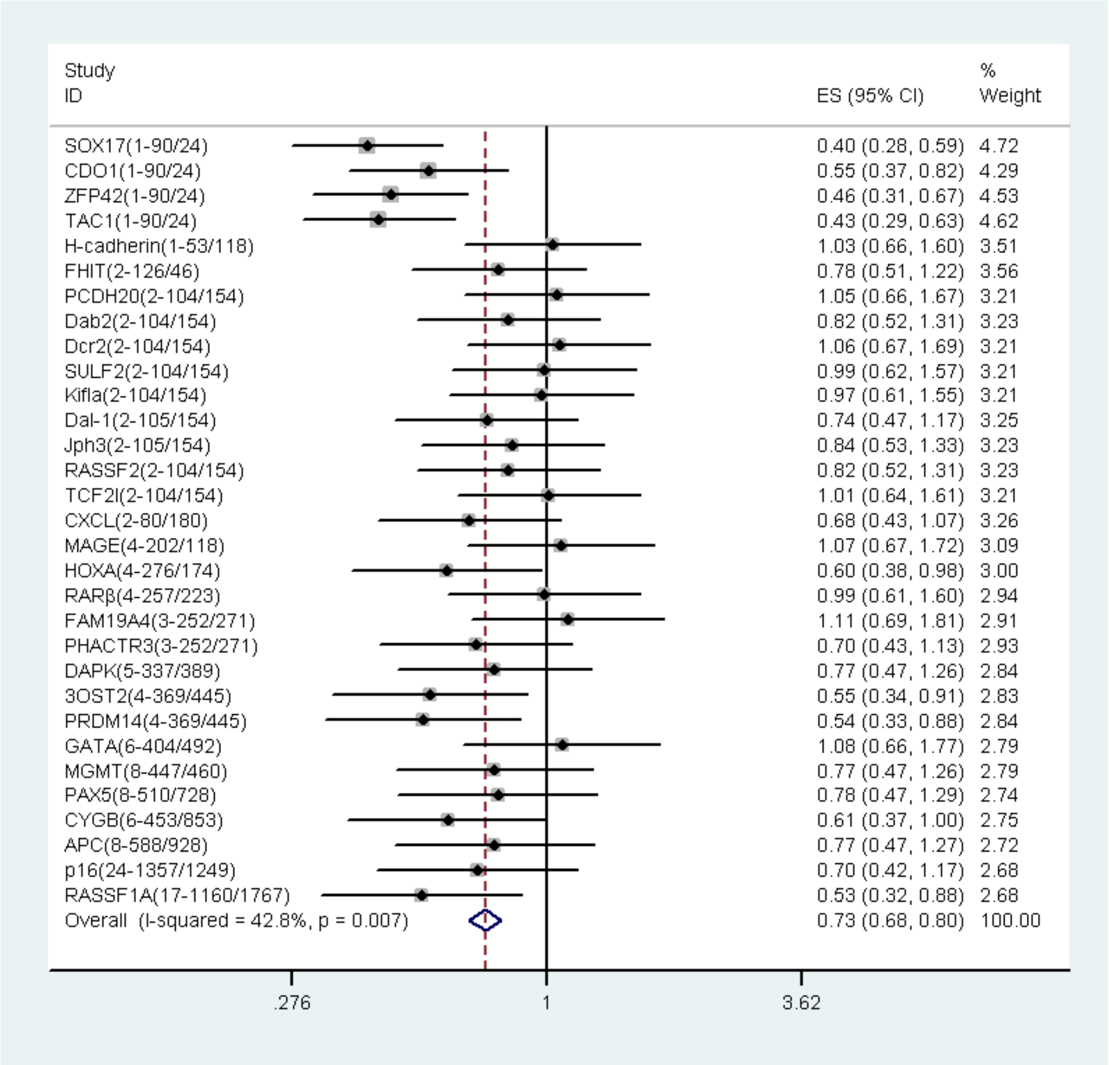

Figure S1. The diagnostic accuracy of 1/*CDH1* compared with the other 31 methylated genes. OR >1 means that 1/*CDH* had a higher diagnostic accuracy, and 95% CI excluding 1 was considered significant.

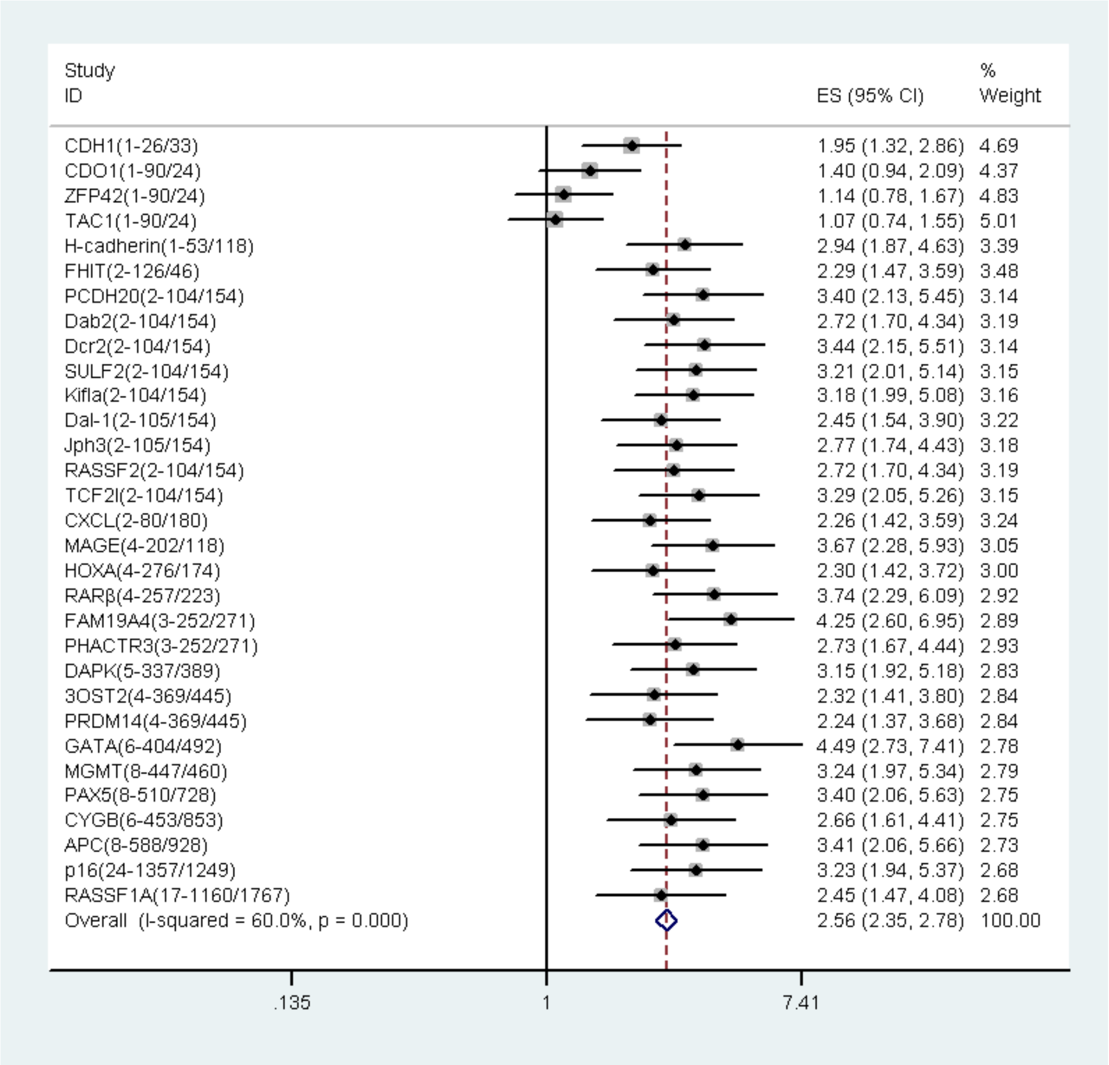

Figure S2. The diagnostic accuracy of 2/*SOX17* compared with the other 31 methylated genes. OR >1 means that 2/*SOX17* had a higher diagnostic accuracy, and 95% CI excluding 1 was considered significant.

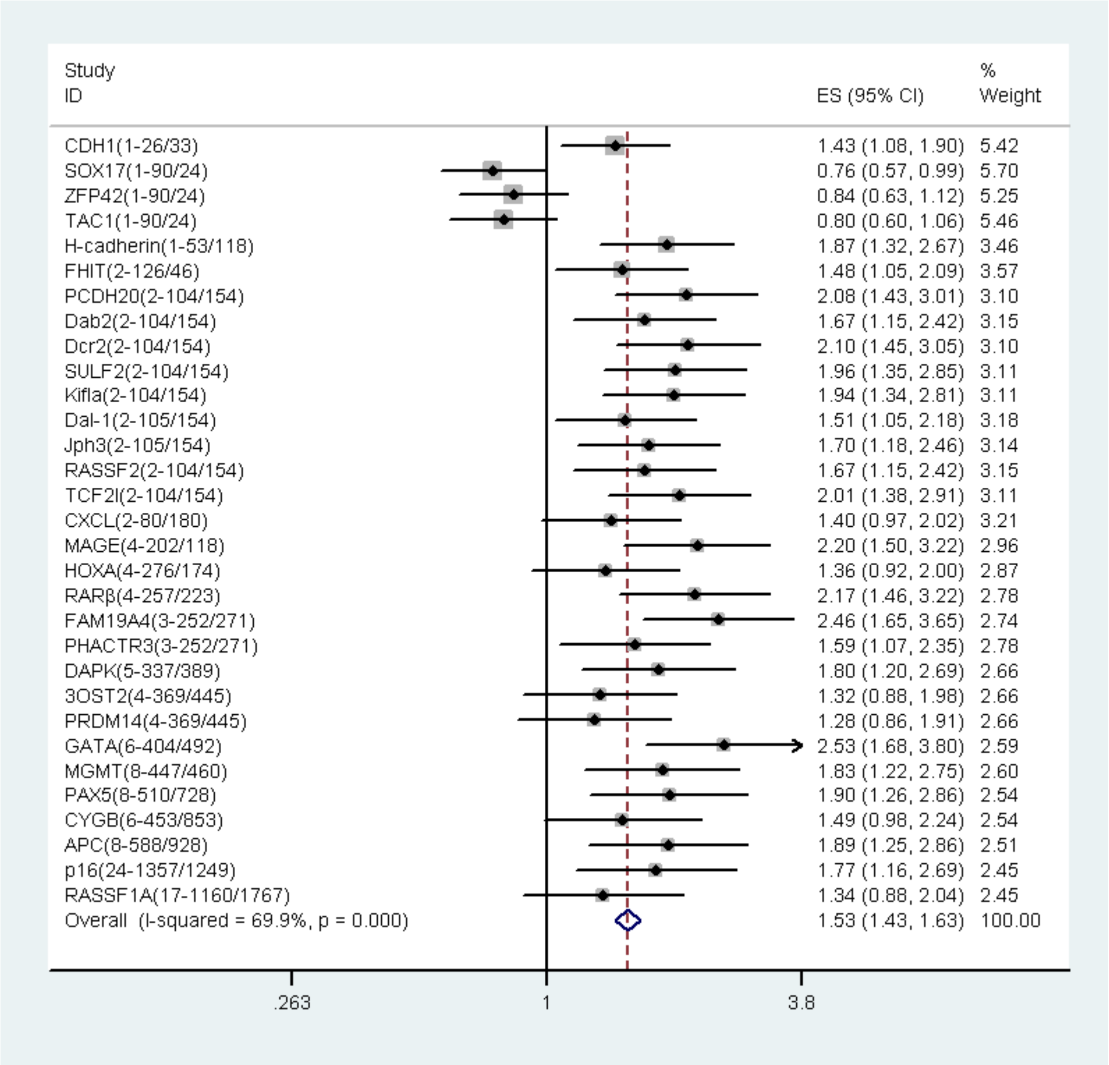

Figure S3. The diagnostic accuracy of 3/*CDO1* compared with the other 31 methylated genes. OR >1 means that 3/*CDO1* had a higher diagnostic accuracy, and 95% CI excluding 1 was considered significant.

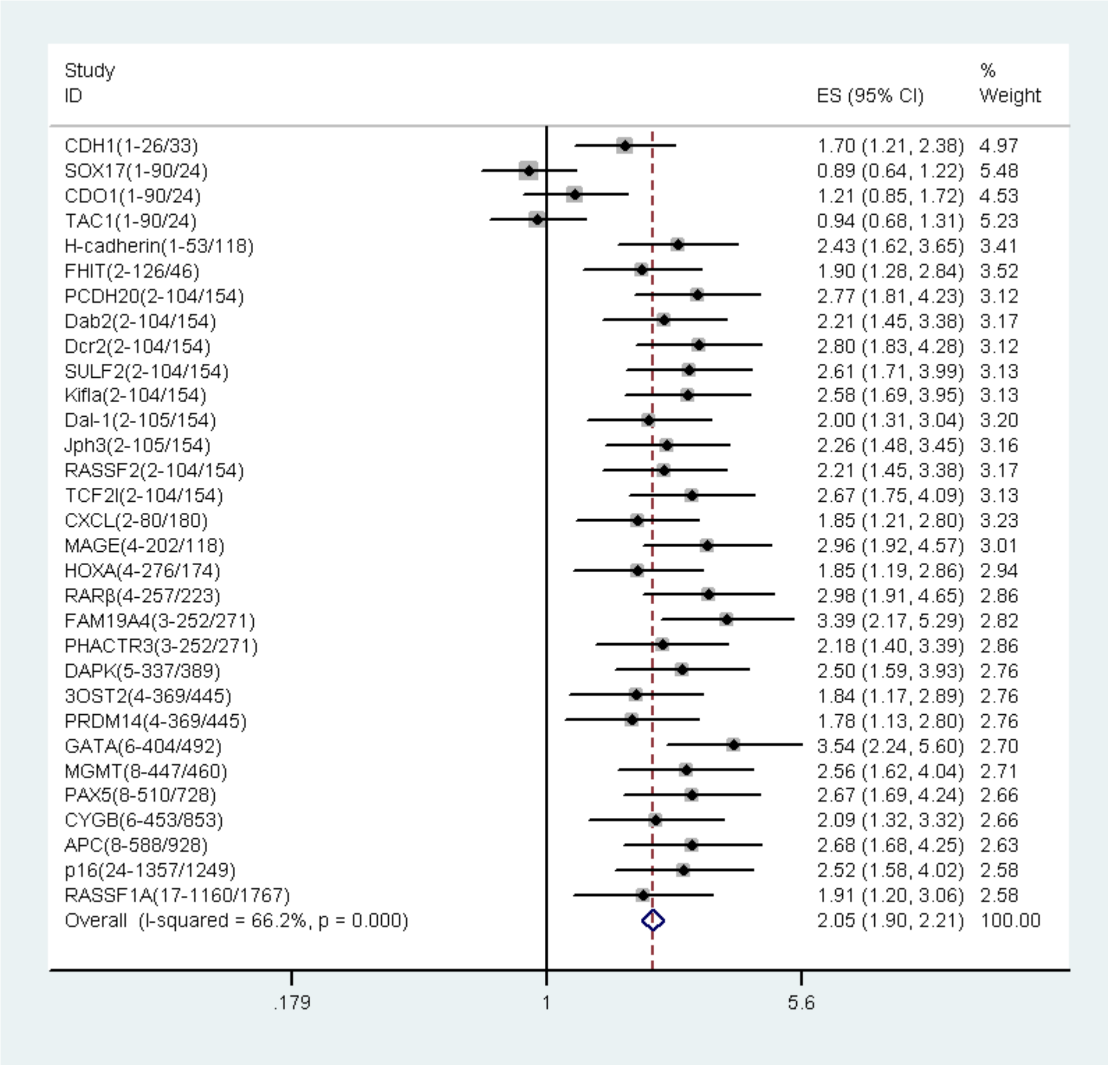

Figure S4. The diagnostic accuracy of 4/*ZFP42* compared with the other 31 methylated genes. OR >1 means that 4/*ZFP42* had a higher diagnostic accuracy, and 95% CI excluding 1 was considered significant.

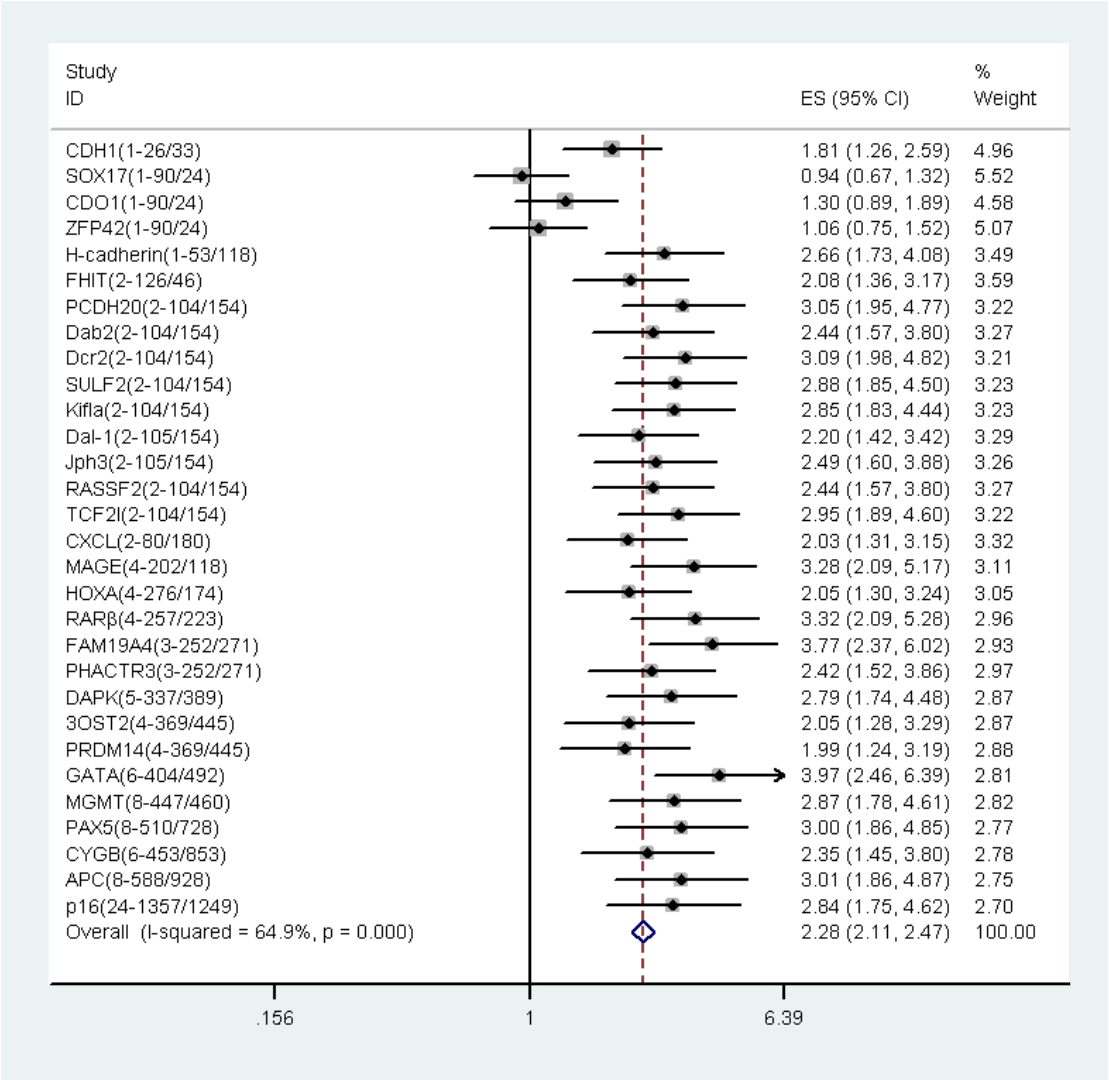

Figure S5. The diagnostic accuracy of 5/*TAC1* compared with the other 28 methylated genes. OR >1 means that 5/*TAC1* had a higher diagnostic accuracy, and 95% CI excluding 1 was considered significant.

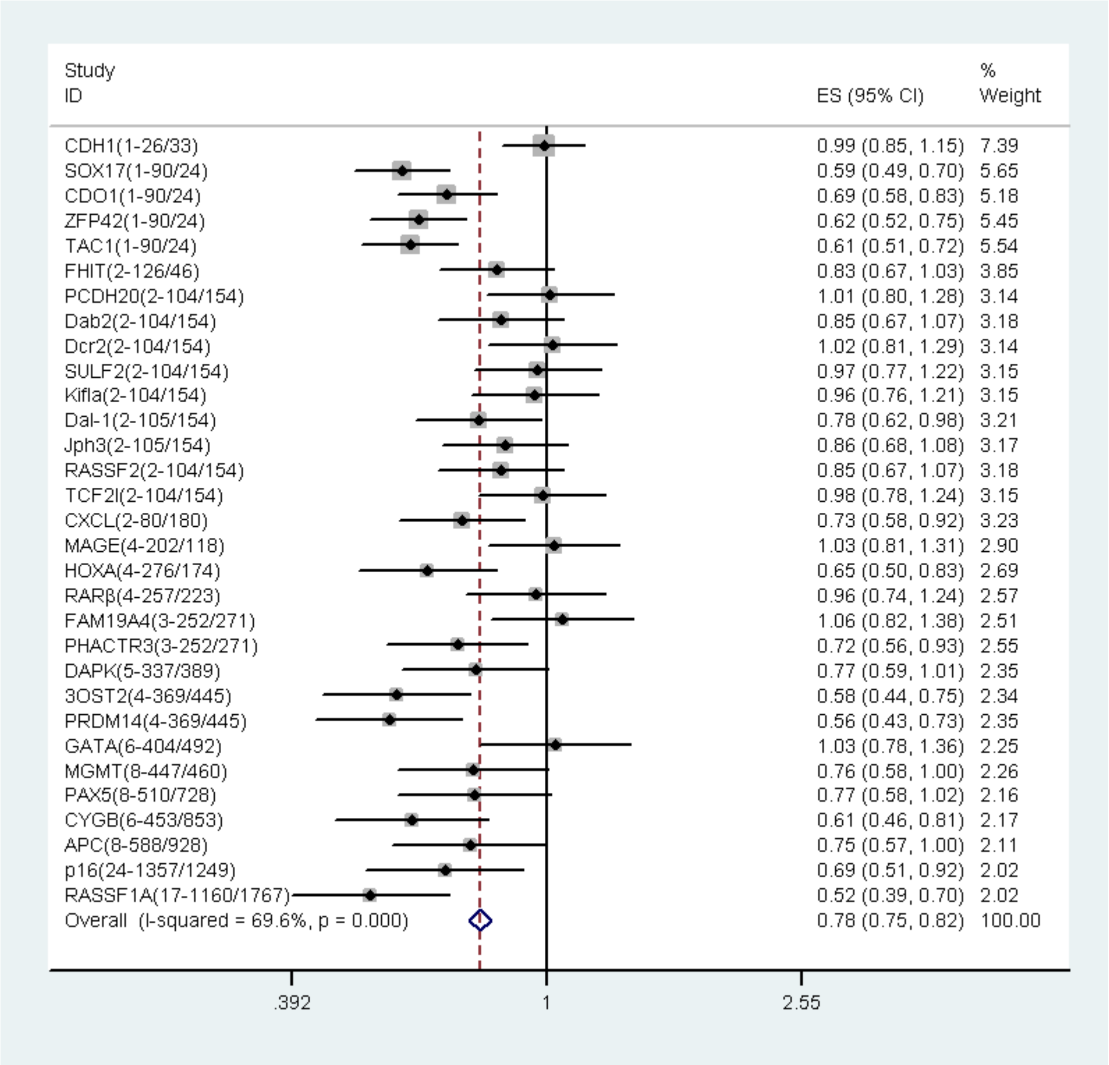

Figure S6. The diagnostic accuracy of 6/*H-cadherin* compared with the other 31 methylated genes. OR >1 means that 6/*H-cadherin* had a higher diagnostic accuracy, and 95% CI excluding 1 was considered significant.

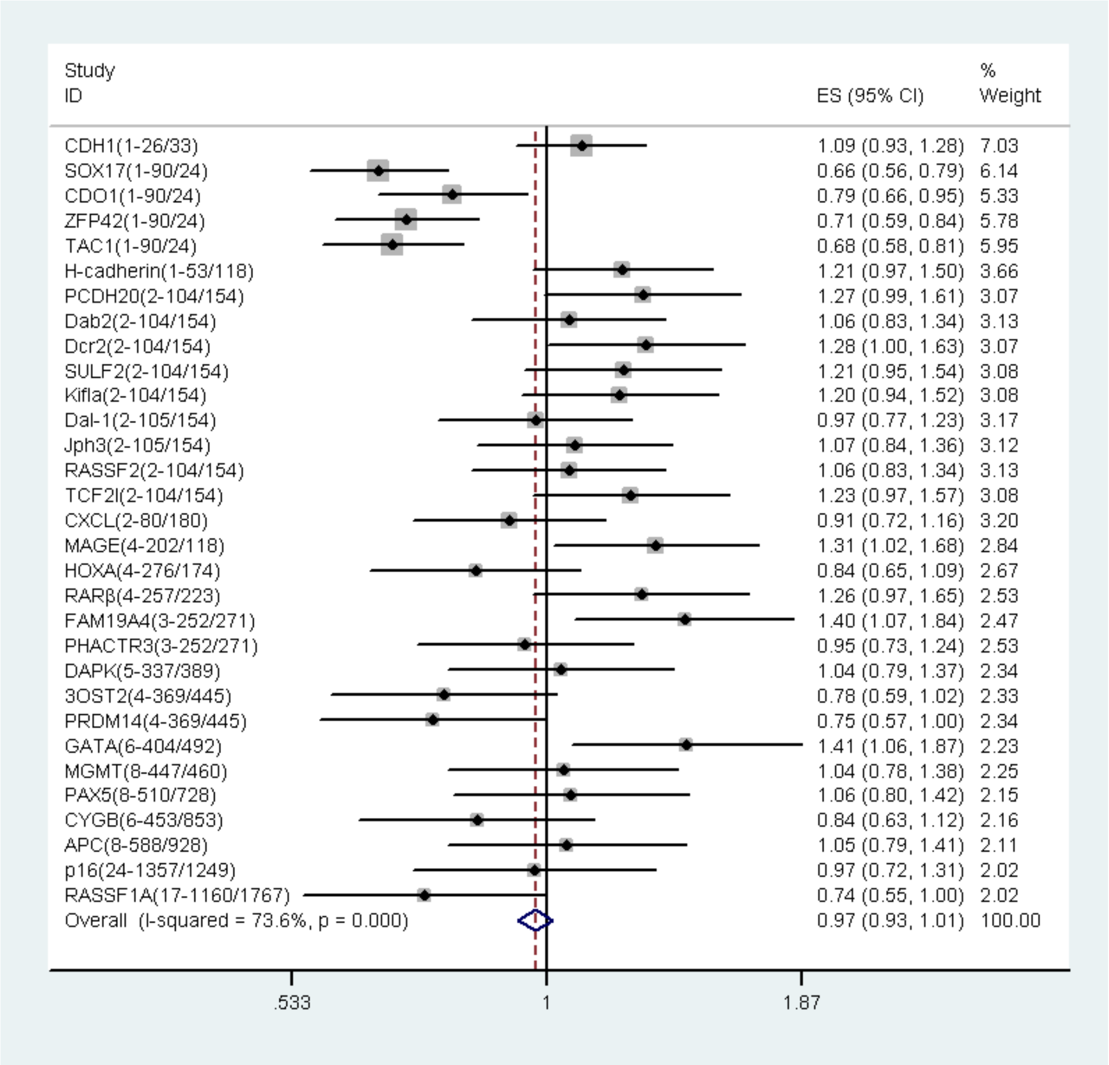

Figure S7. The diagnostic accuracy of 7/*FHIT* compared with the other 31 methylated genes. OR >1 means that 7/*FHIT* had a higher diagnostic accuracy, and 95% CI excluding 1 was considered significant.

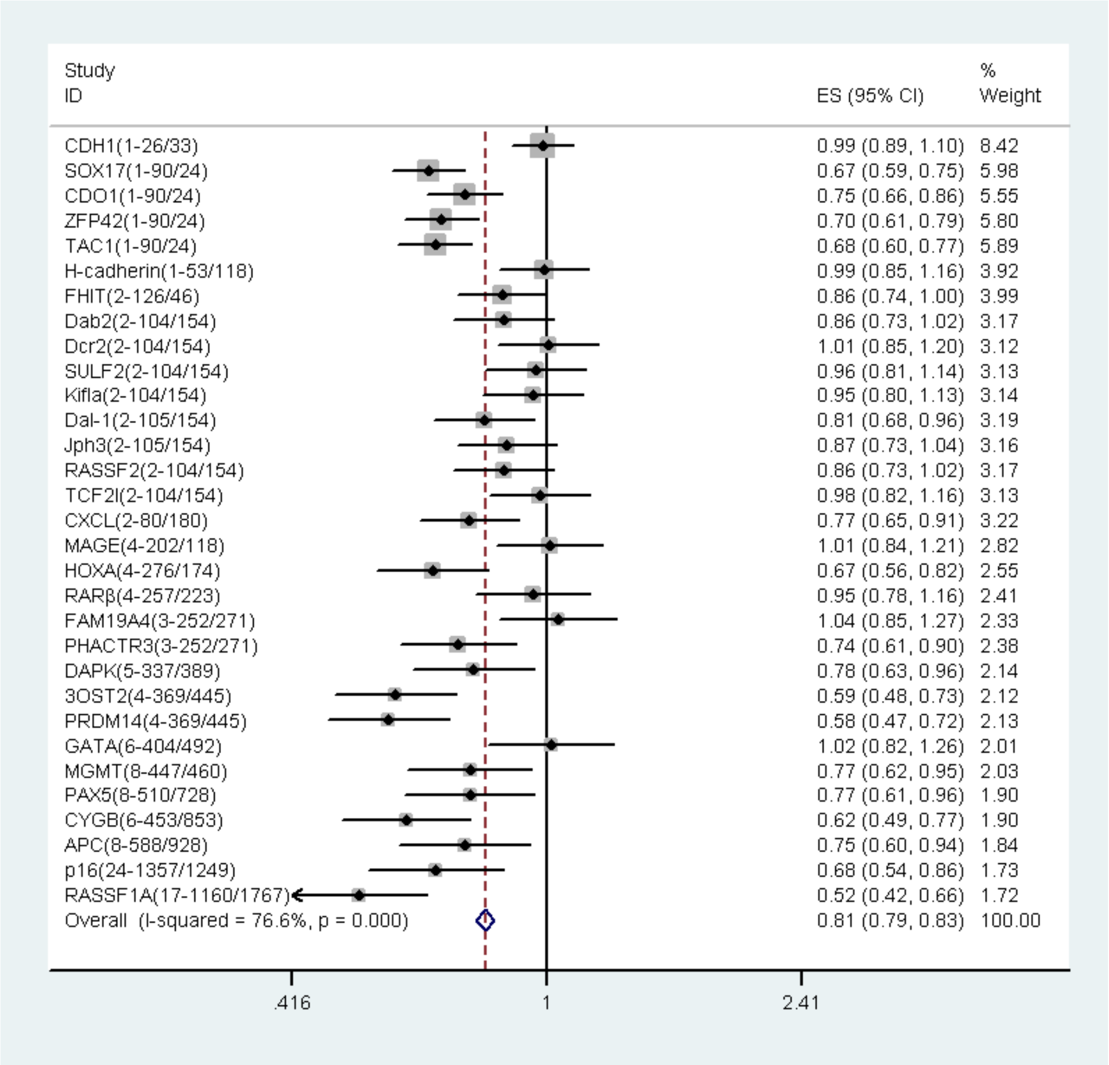

Figure S8. The diagnostic accuracy of 8/*PCDH20* compared with the other 31 methylated genes. OR >1 means that 8/*PCDH20* had a higher diagnostic accuracy, and 95% CI excluding 1 was considered significant.

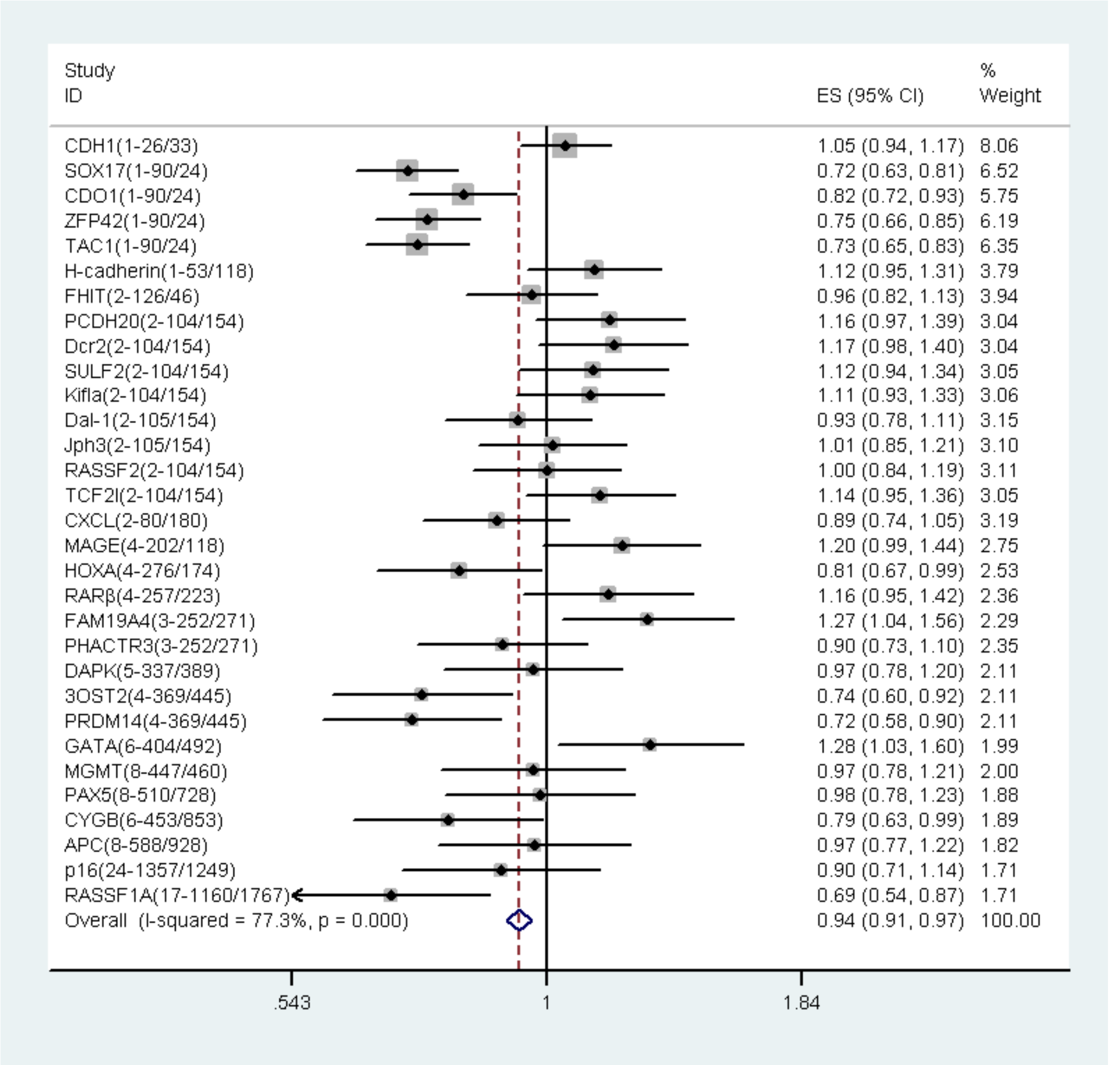

Figure S9. The diagnostic accuracy of 9/*Dab2* compared with the other 31 methylated genes. OR >1 means that 9/*Dab2* had a higher diagnostic accuracy, and 95% CI excluding 1 was considered significant.

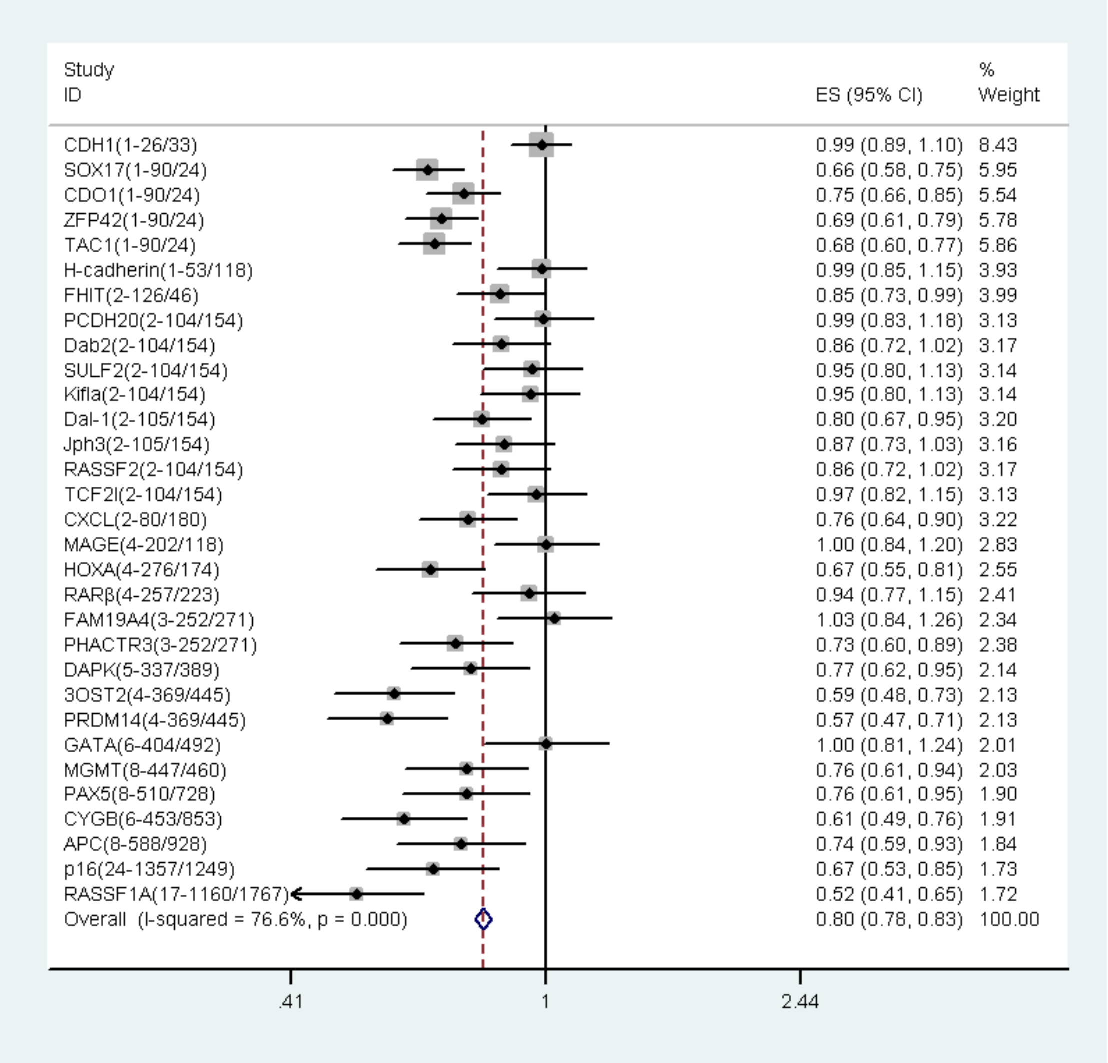

Figure S10. The diagnostic accuracy of 10/*Dcr2* compared with the other 31 methylated genes. OR >1 means that 10/*Dcr2* had a higher diagnostic accuracy, and 95% CI excluding 1 was considered significant.

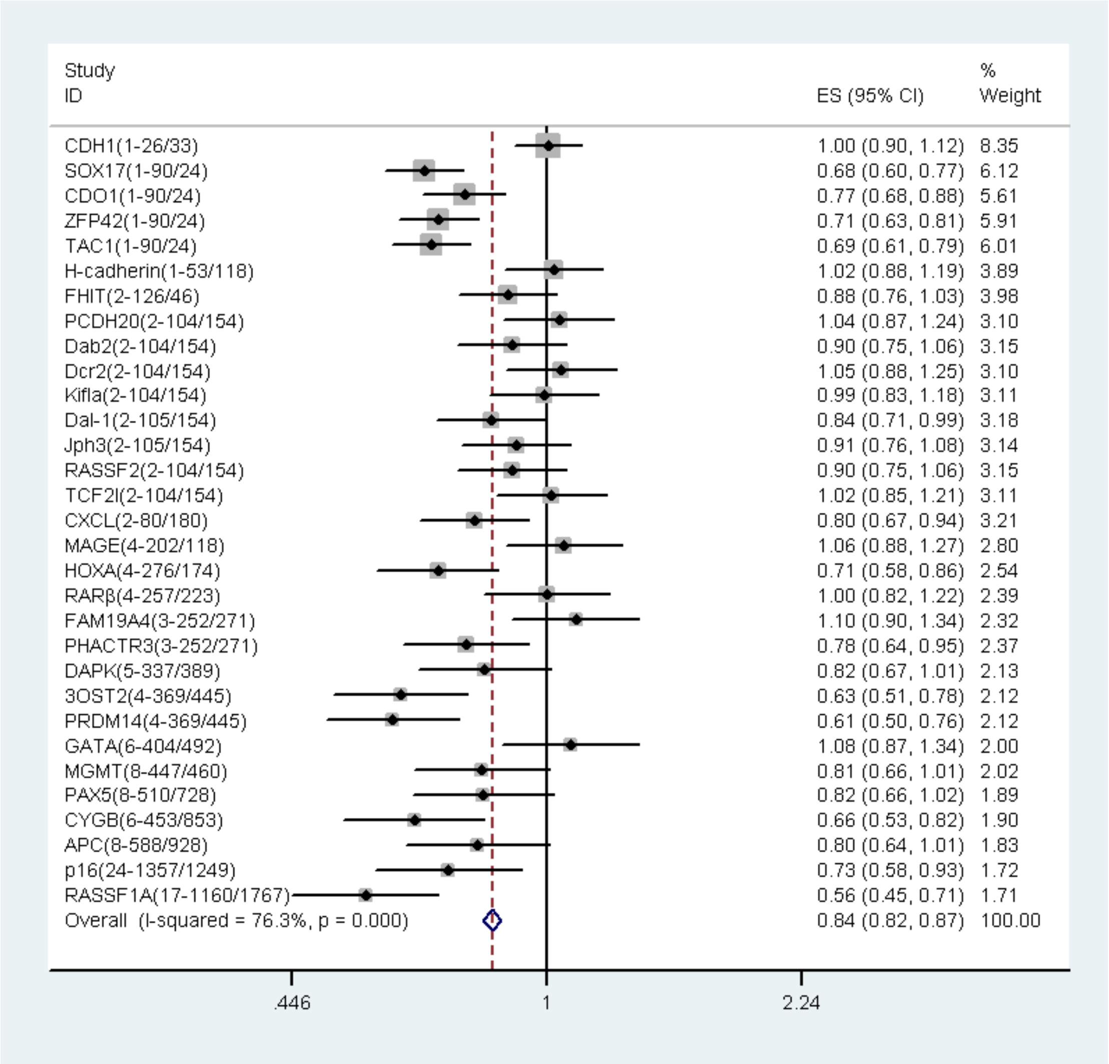

Figure S11. The diagnostic accuracy of 11/*SULF2* compared with the other 31 methylated genes. OR >1 means that 11/*SULF2* had a higher diagnostic accuracy, and 95% CI excluding 1 was considered significant.

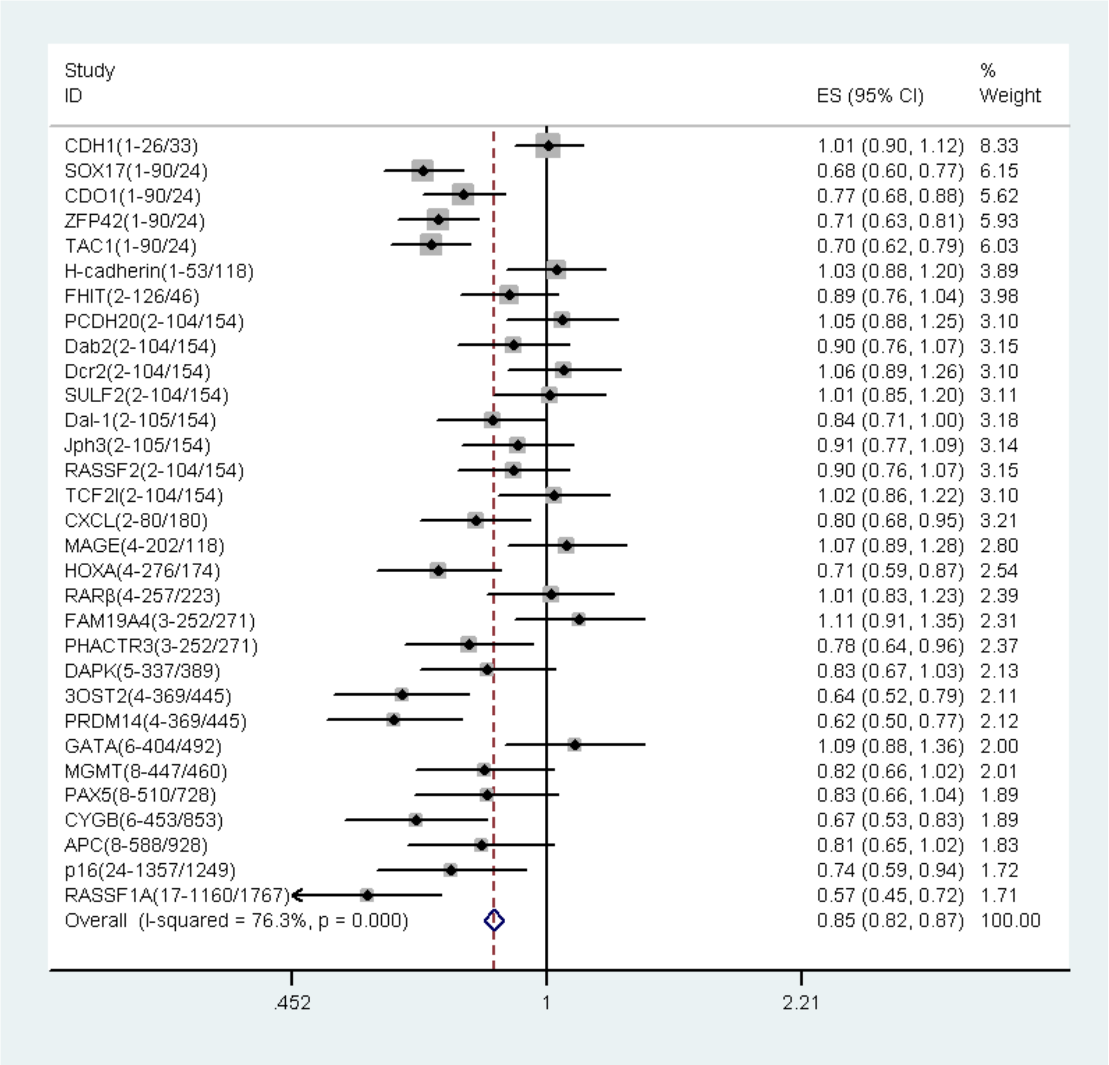

Figure S12. The diagnostic accuracy of 12/*Kifla* compared with the other 31 methylated genes. OR >1 means that 12/*Kifla* had a higher diagnostic accuracy, and 95% CI excluding 1 was considered significant.

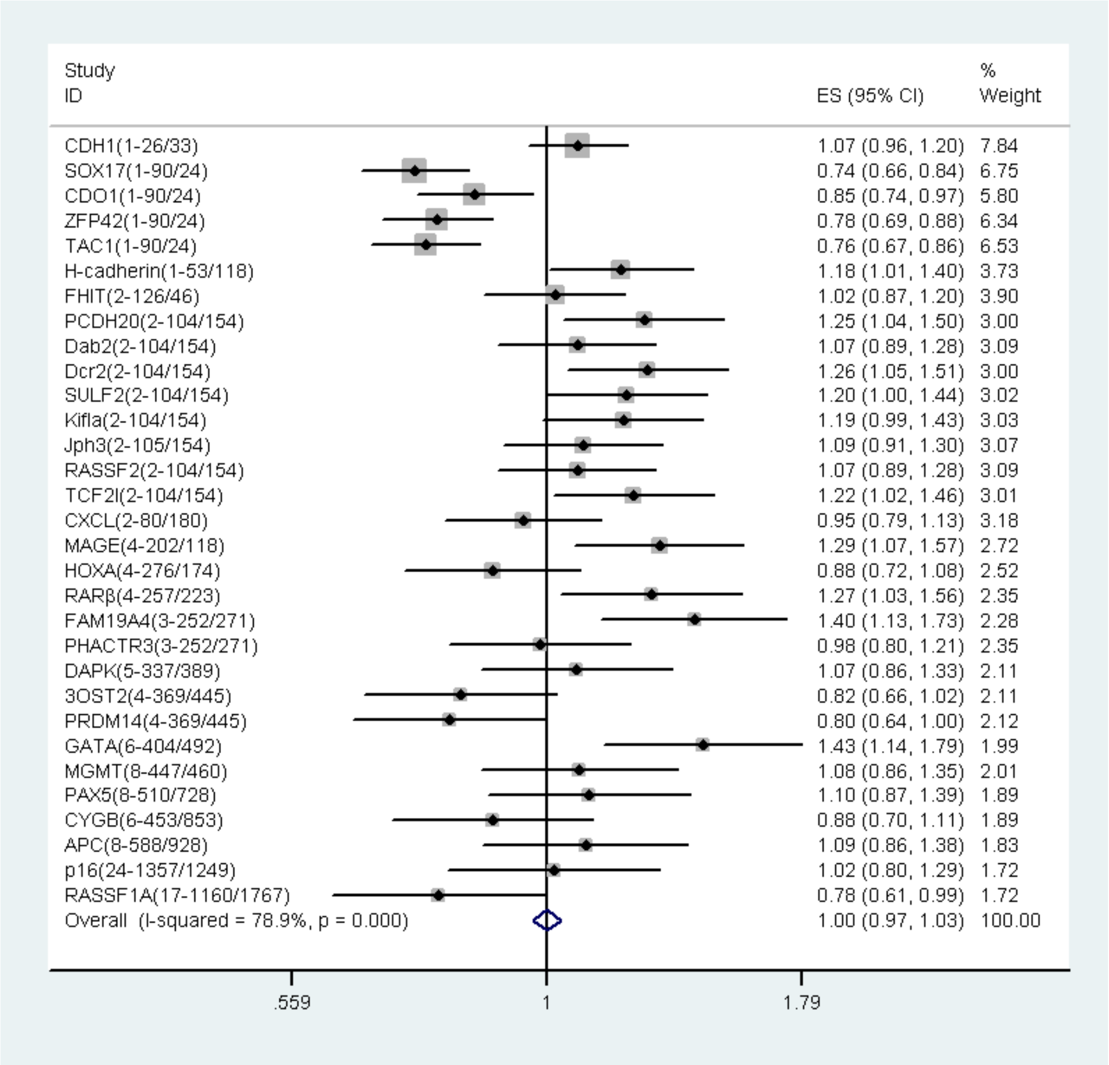

Figure S13. The diagnostic accuracy of 13/*Dal-1* compared with the other 31 methylated genes. OR >1 means that 13/*Dal-1* had a higher diagnostic accuracy, and 95% CI excluding 1 was considered significant.

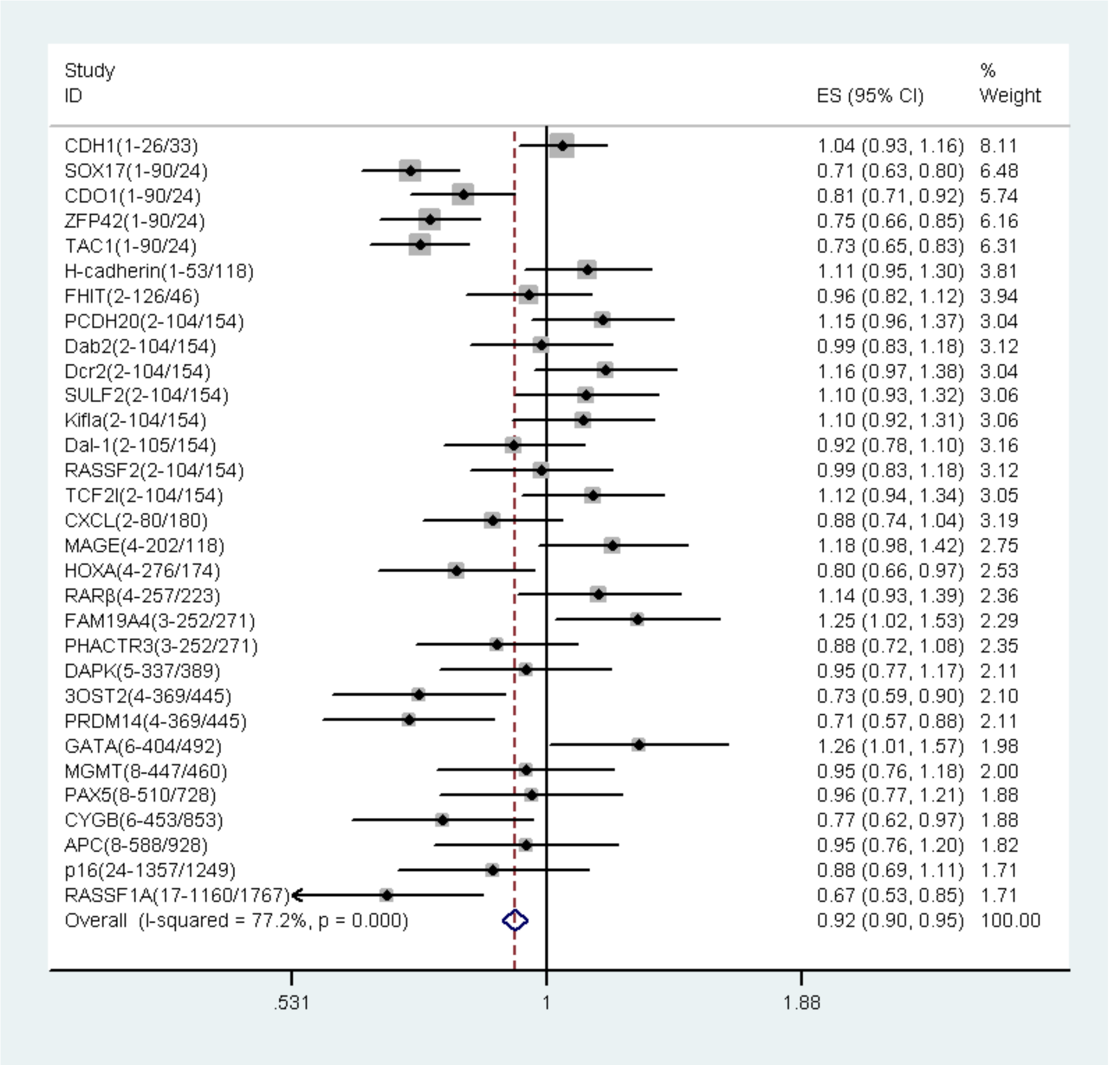

Figure S14. The diagnostic accuracy of 14/*Jph3* compared with the other 31 methylated genes. OR >1 means that 14/*Jph3* had a higher diagnostic accuracy, and 95% CI excluding 1 was considered significant.

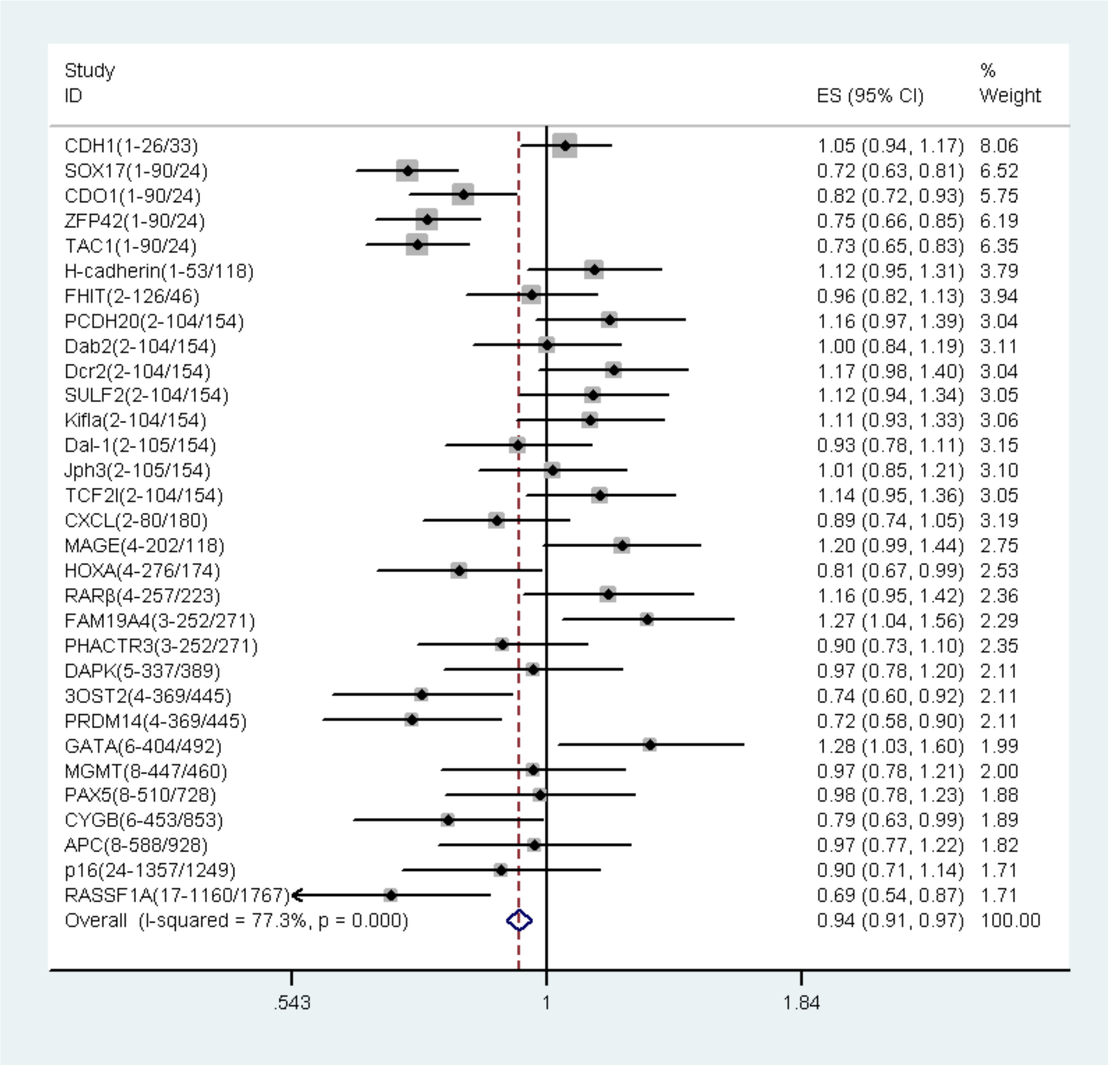

Figure S15. The diagnostic accuracy of 15/*RASSF2* compared with the other 31 methylated genes. OR >1 means that 15/*RASSF2* had a higher diagnostic accuracy, and 95% CI excluding 1 was considered significant.

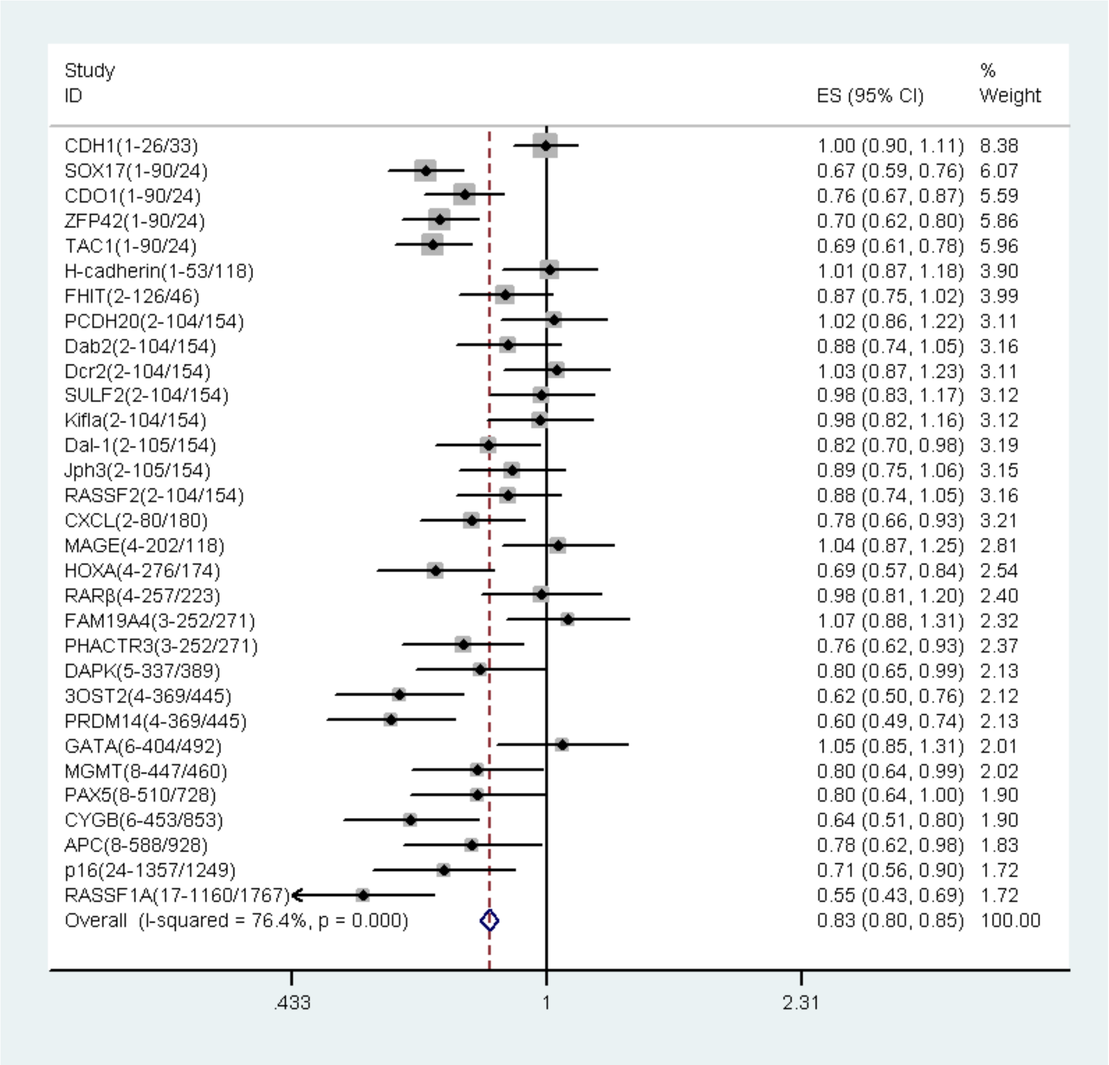

Figure S16. The diagnostic accuracy of 16/*TCF2l* compared with the other 31 methylated genes. OR >1 means that 16/*TCF2l* had a higher diagnostic accuracy, and 95% CI excluding 1 was considered significant.

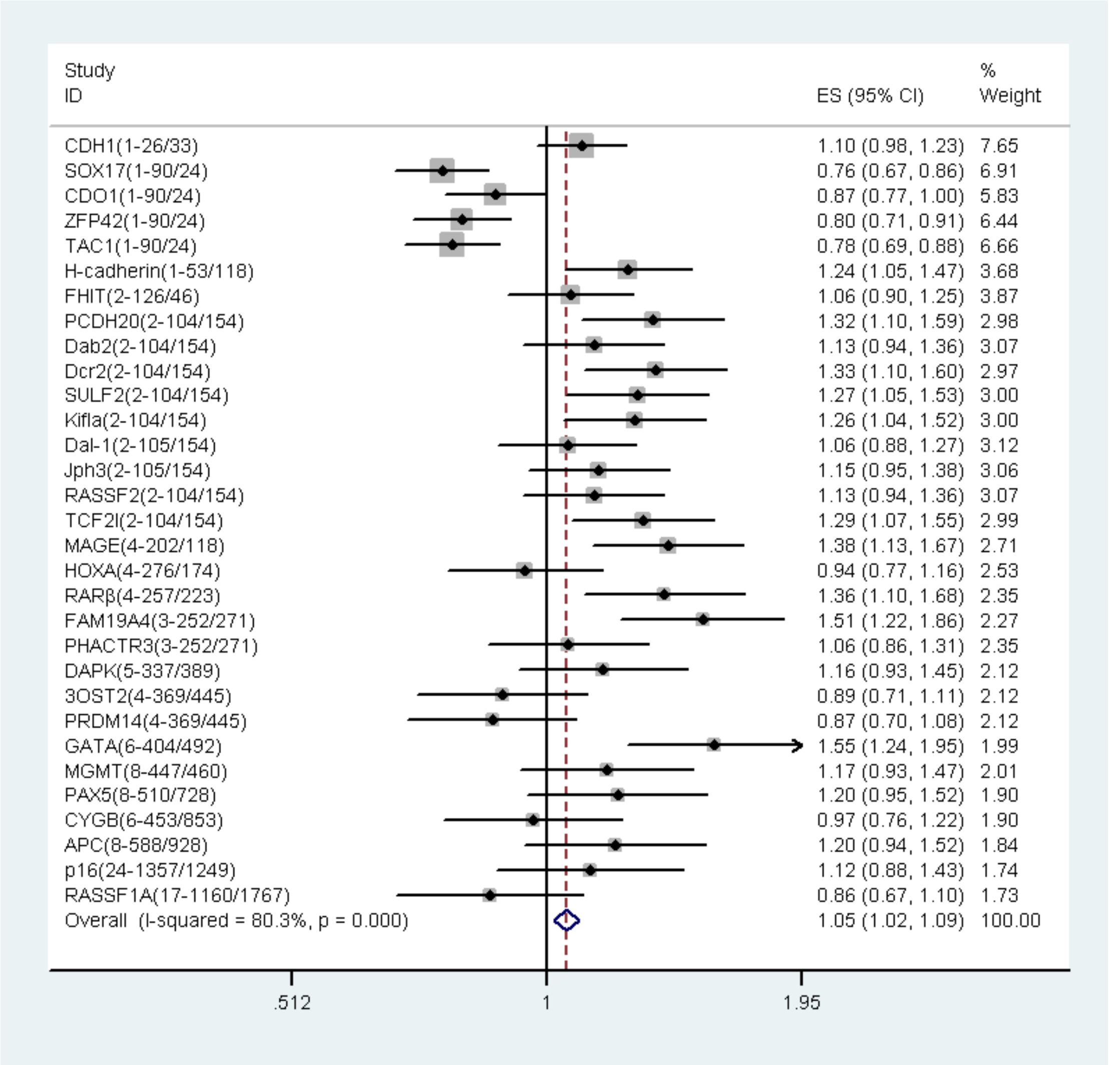

Figure S17. The diagnostic accuracy of 17/*CXCL* compared with the other 31 methylated genes. OR >1 means that 17/*CXCL* had a higher diagnostic accuracy, and 95% CI excluding 1 was considered significant.

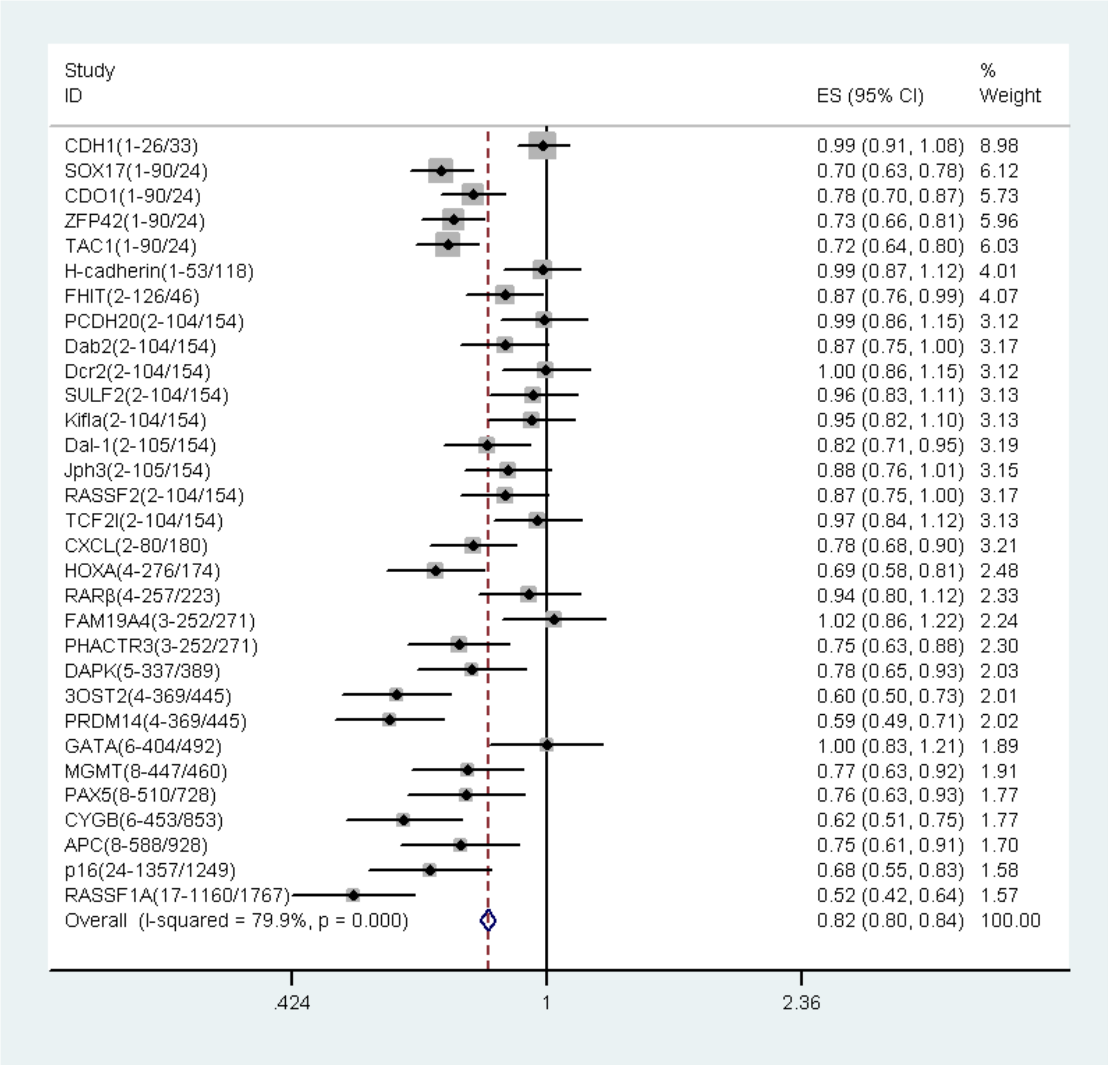

Figure S18. The diagnostic accuracy of 18/*MAGE* compared with the other 31 methylated genes. OR >1 means that 18/*MAGE* had a higher diagnostic accuracy, and 95% CI excluding 1 was considered significant.

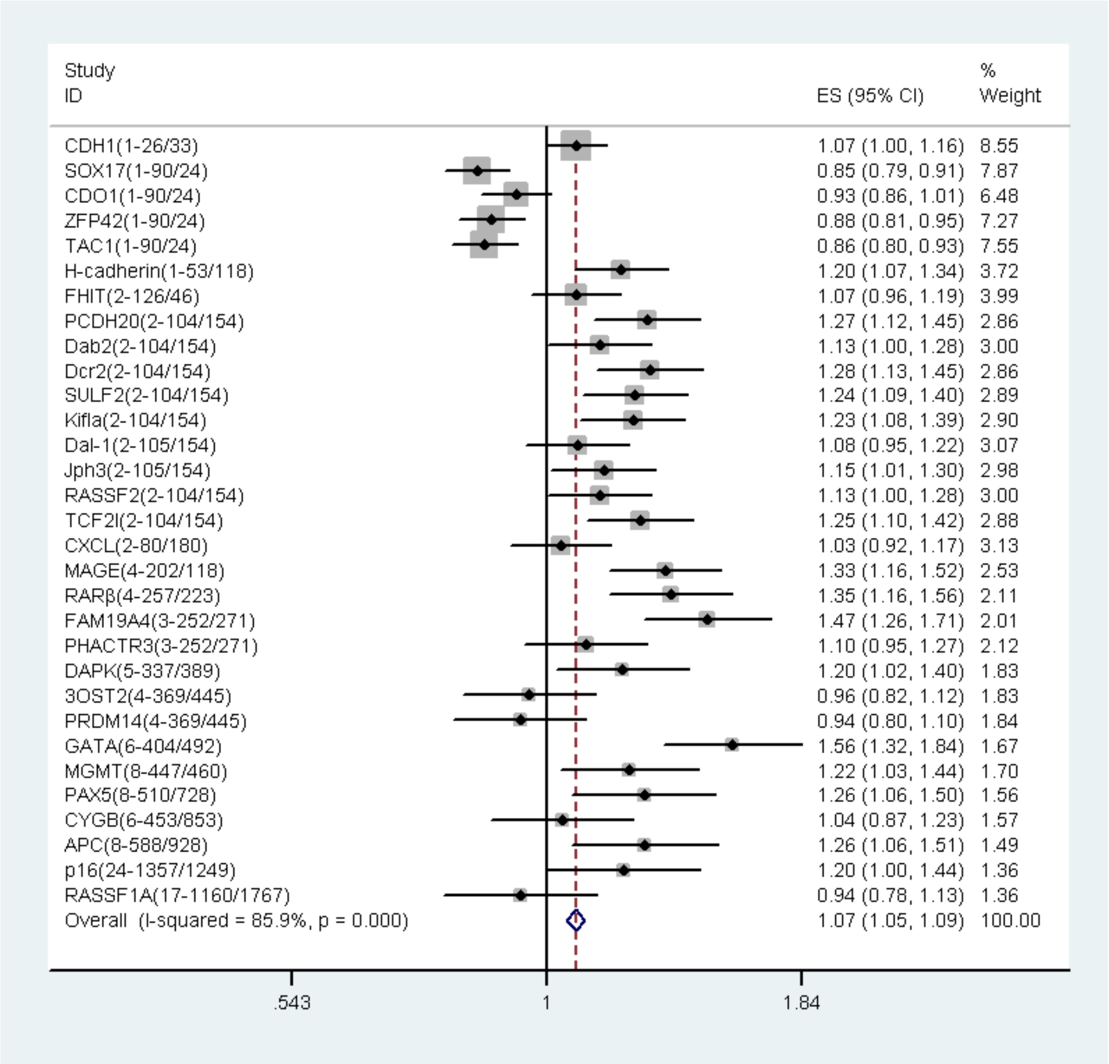

Figure S19. The diagnostic accuracy of 19/*HOXA* compared with the other 31 methylated genes. OR >1 means that 19/*HOXA* had a higher diagnostic accuracy, and 95% CI excluding 1 was considered significant.

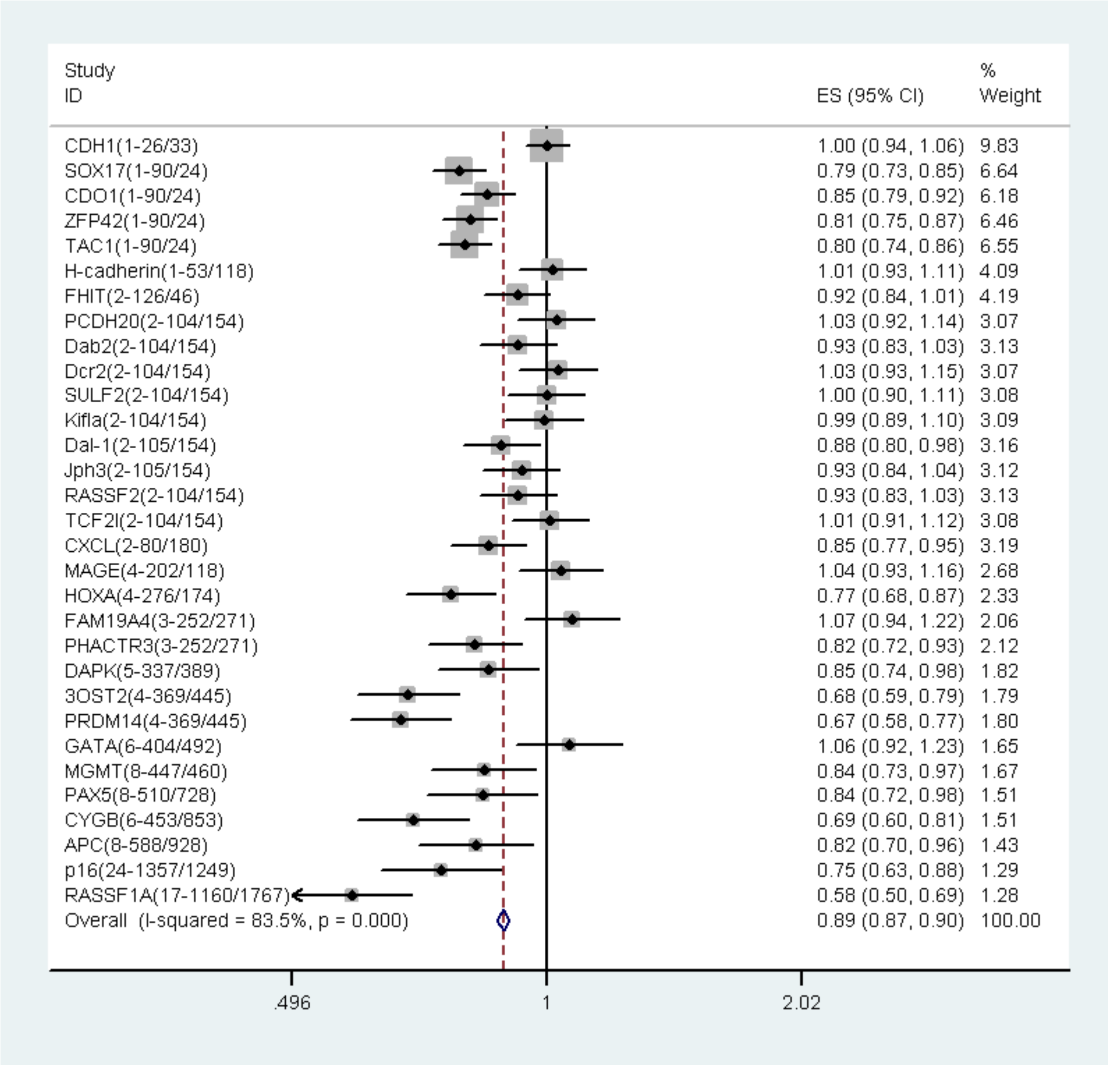

Figure S20. The diagnostic accuracy of 20/*RARβ* compared with the other 31 methylated genes. OR >1 means that 20/*RARβ* had a higher diagnostic accuracy, and 95% CI excluding 1 was considered significant.

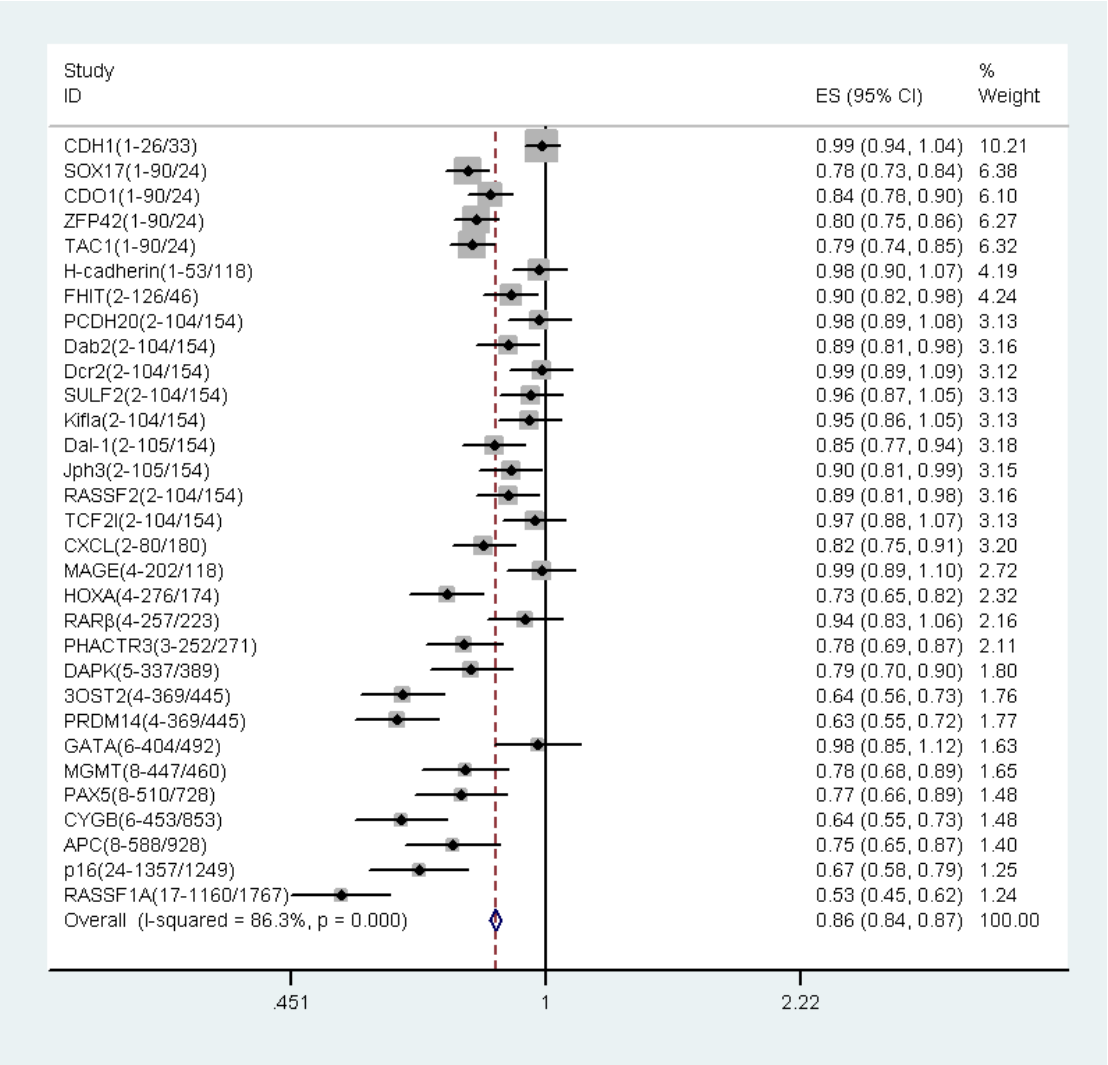

Figure S21. The diagnostic accuracy of 21/*FAM19A4* compared with the other 31 methylated genes. OR >1 means that 21/*FAM19A4* had a higher diagnostic accuracy, and 95% CI excluding 1 was considered significant.

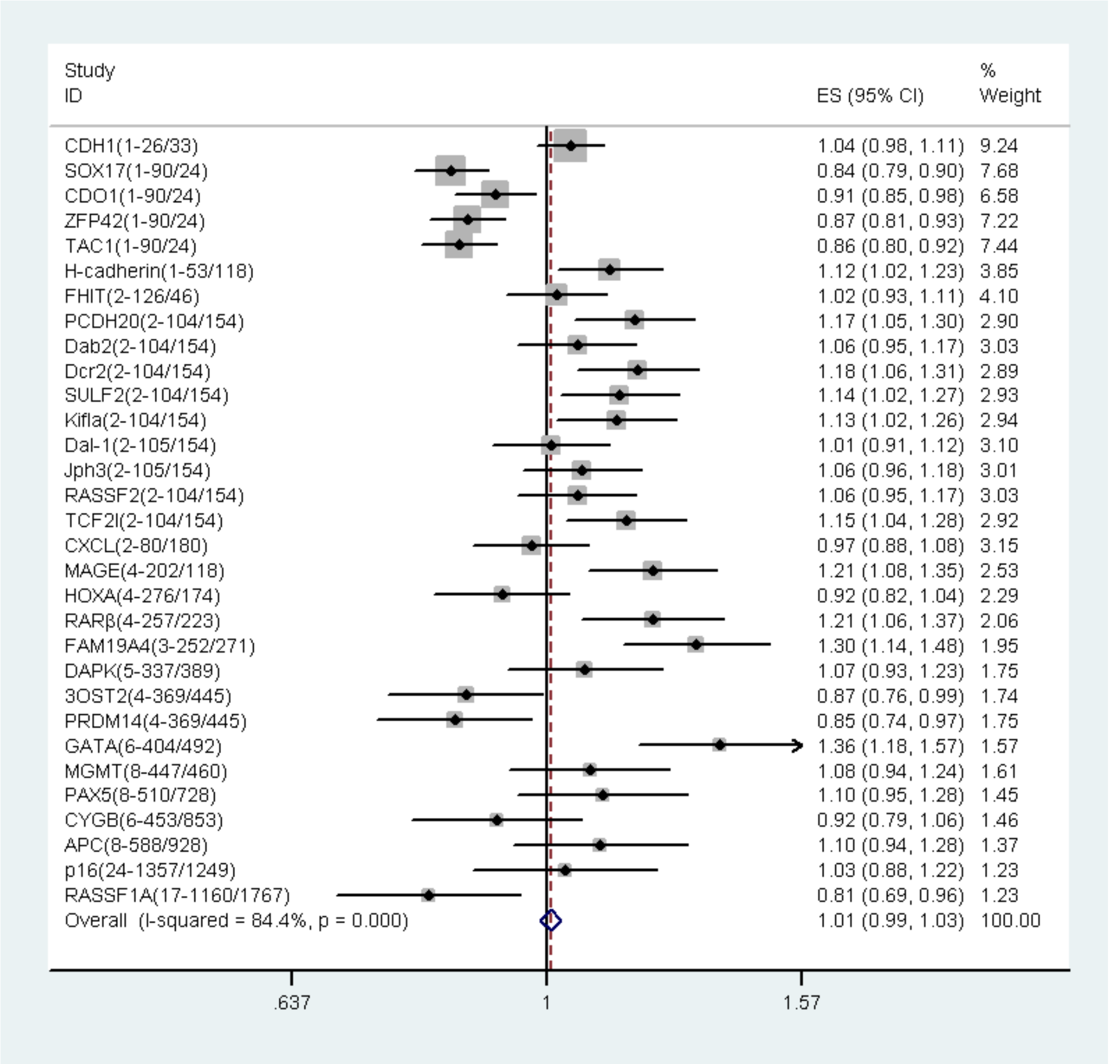

Figure S22. The diagnostic accuracy of 22/*PHACTR3* compared with the other 31 methylated genes. OR >1 means that 22/*PHACTR3* had a higher diagnostic accuracy, and 95% CI excluding 1 was considered significant.

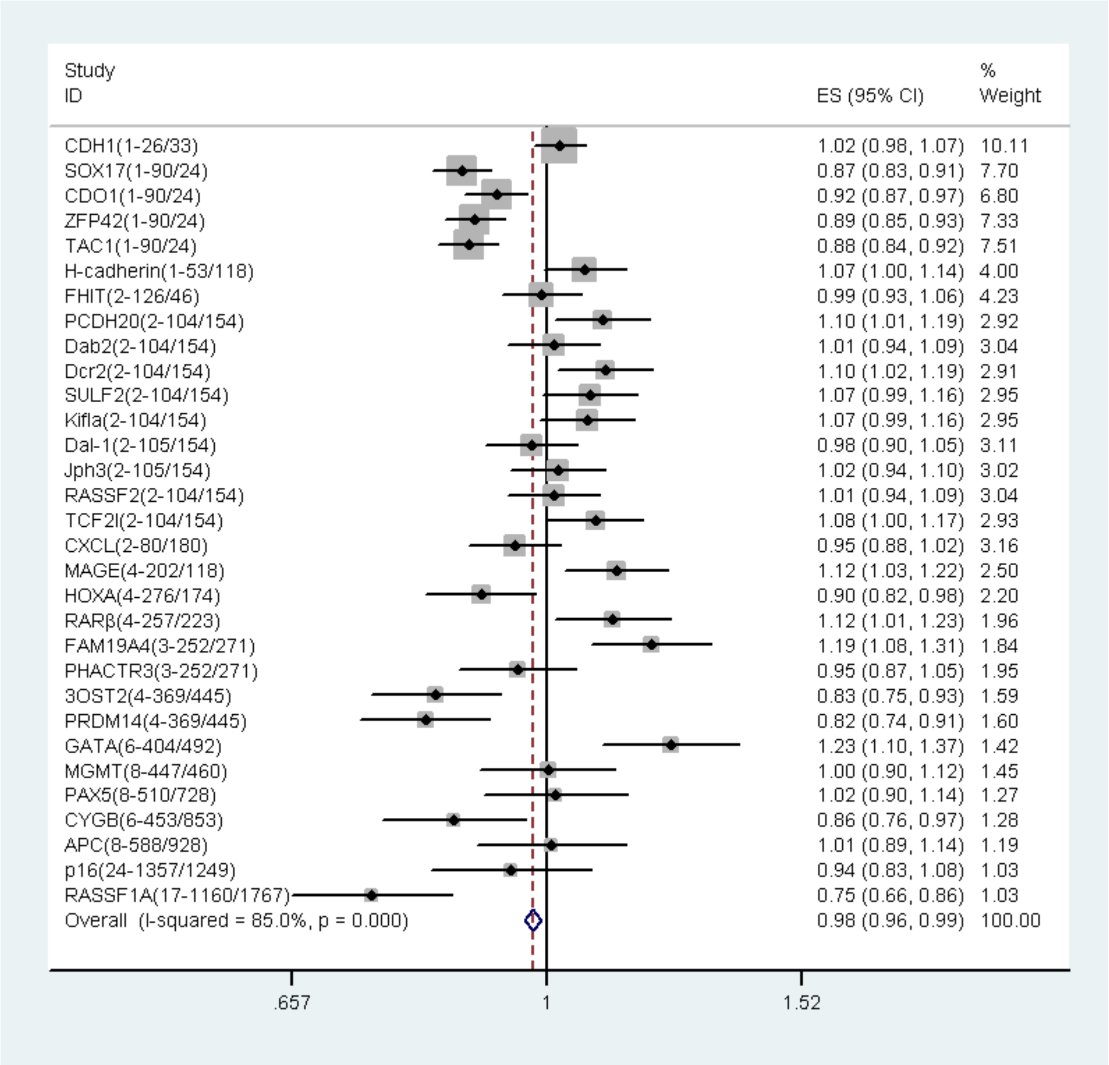

Figure S23. The diagnostic accuracy of 23/*DAPK* compared with the other 31 methylated genes. OR >1 means that 23/*DAPK* had a higher diagnostic accuracy, and 95% CI excluding 1 was considered significant.

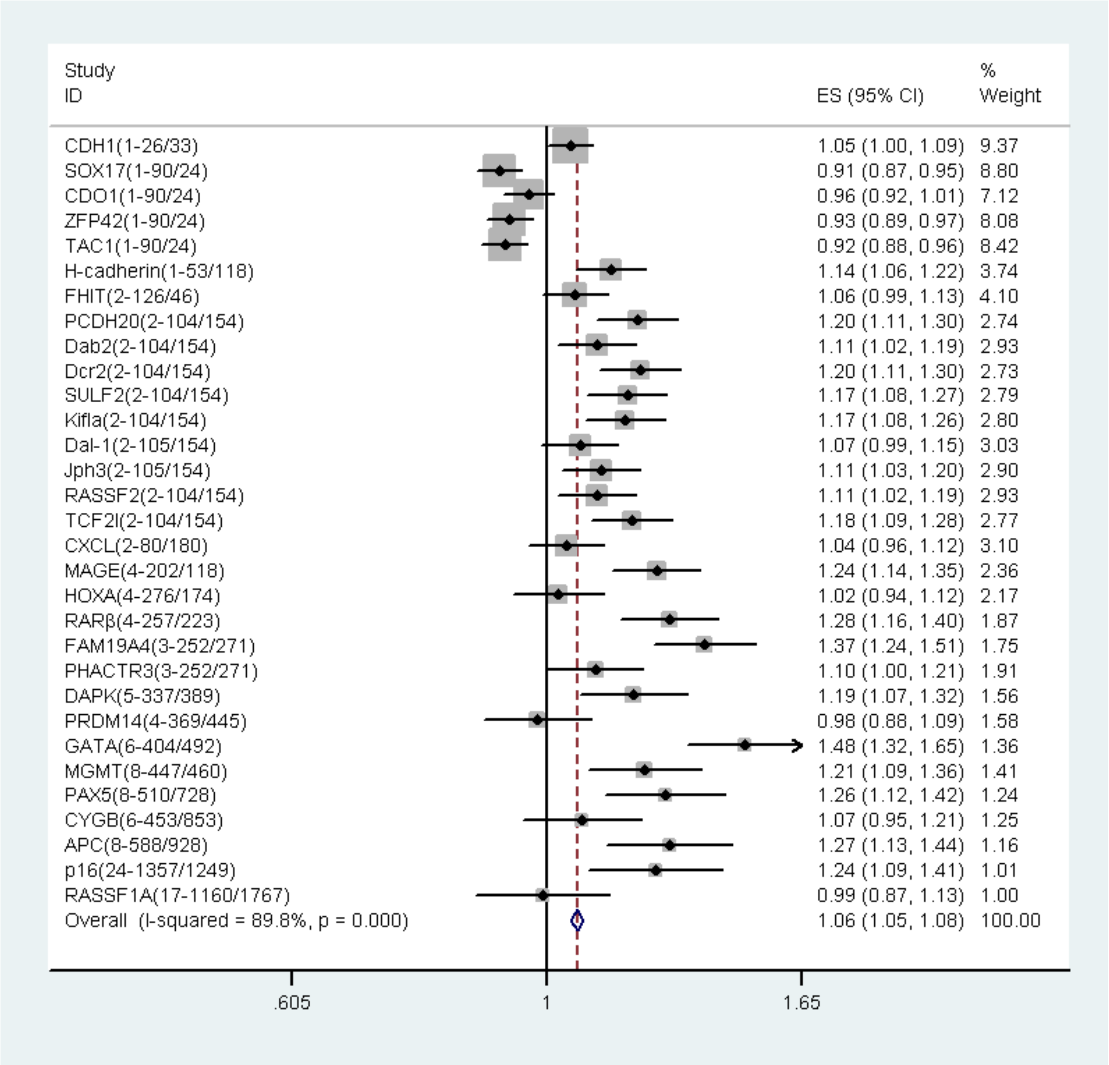

Figure S24. The diagnostic accuracy of 24/*3OST2* compared with the other 28 methylated genes. OR >1 means that 24/*3OST2* had a higher diagnostic accuracy, and 95% CI excluding 1 was considered significant.

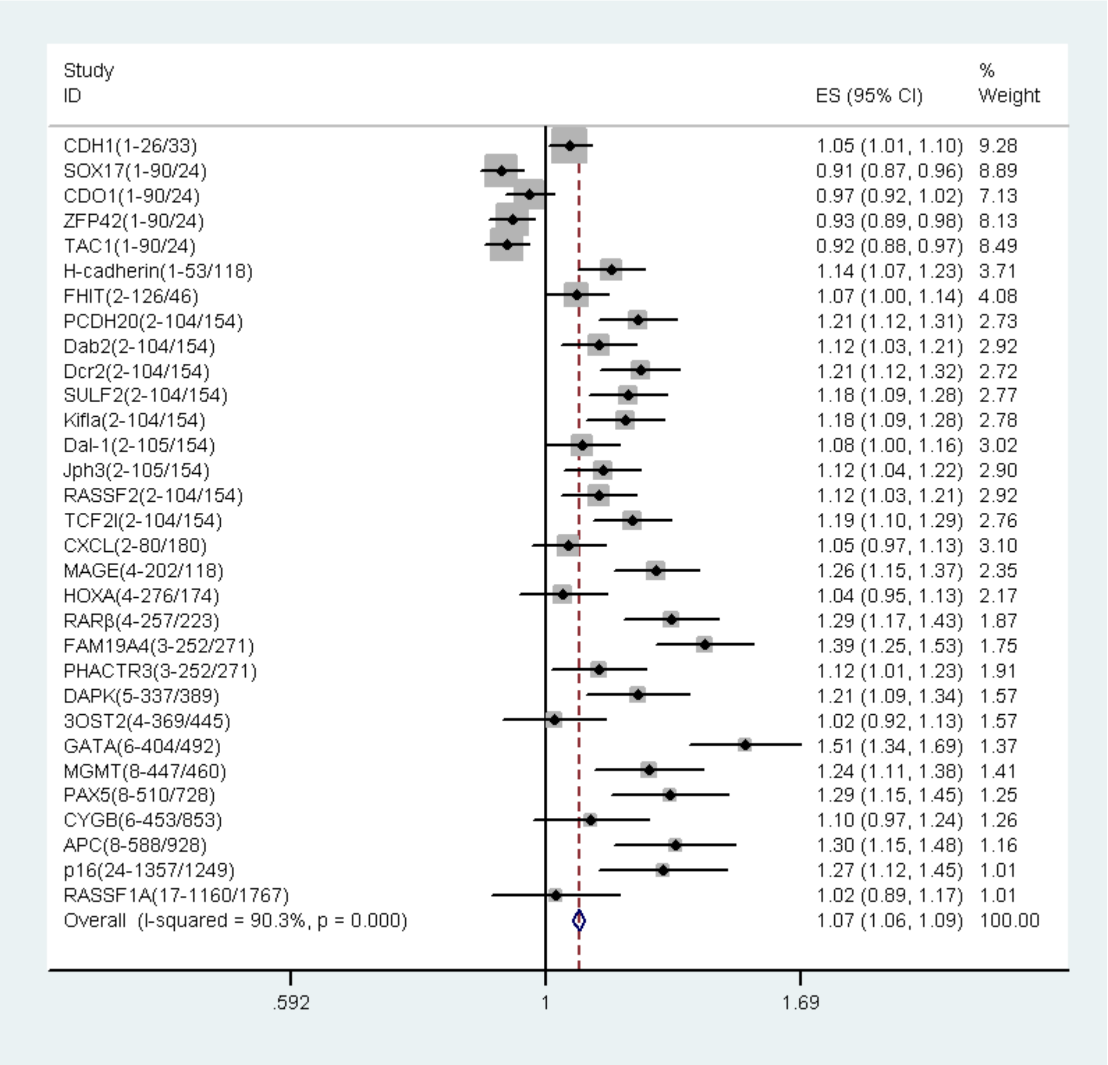

Figure S25. The diagnostic accuracy of 25/*PRDM14* compared with the other 31 methylated genes. OR >1 means that 25/*PRDM14* had a higher diagnostic accuracy, and 95% CI excluding 1 was considered significant.

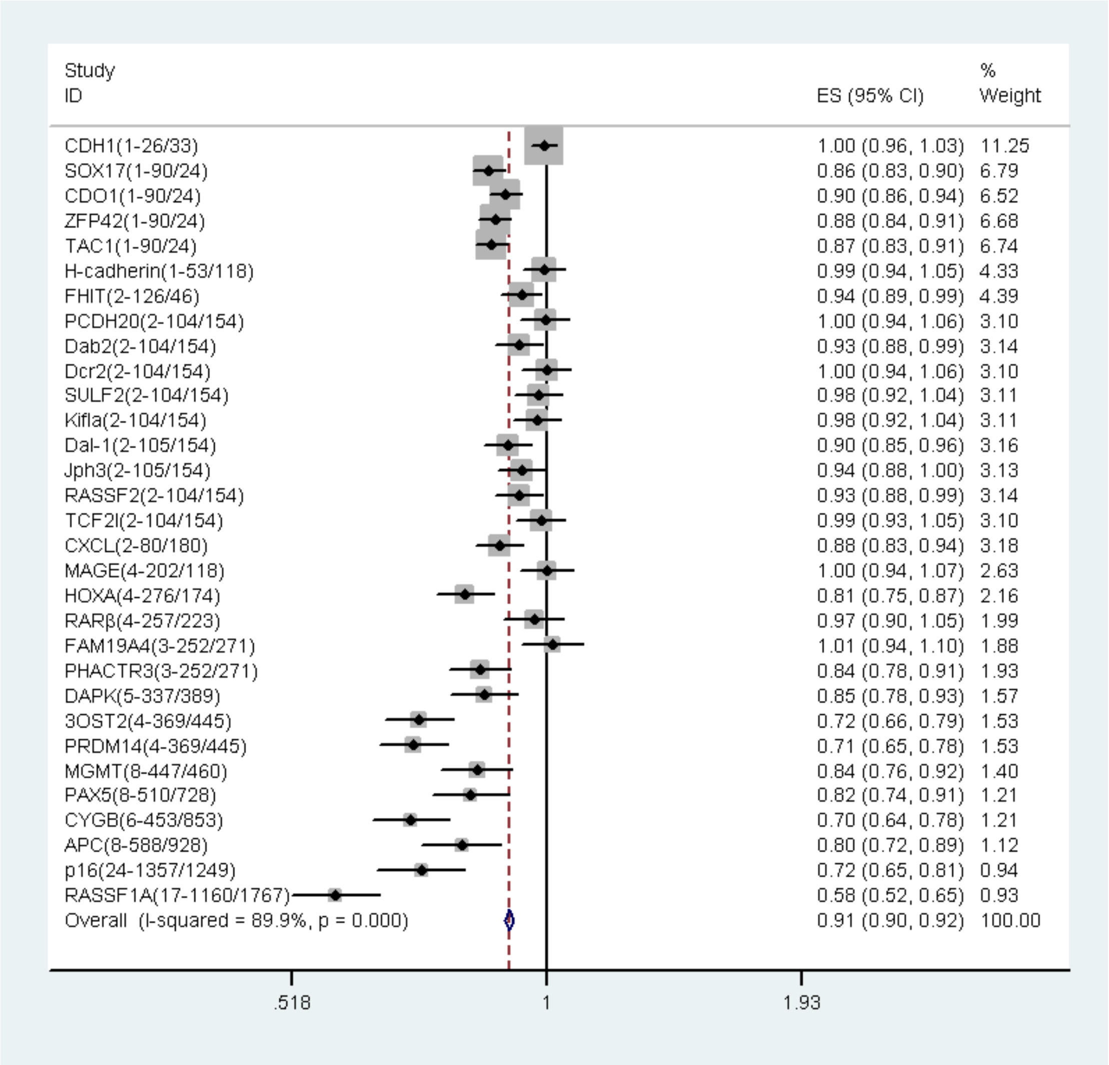

Figure S26. The diagnostic accuracy of 26/*GATA* compared with the other 31 methylated genes. OR >1 means that 31/*GATA* had a higher diagnostic accuracy, and 95% CI excluding 1 was considered significant.

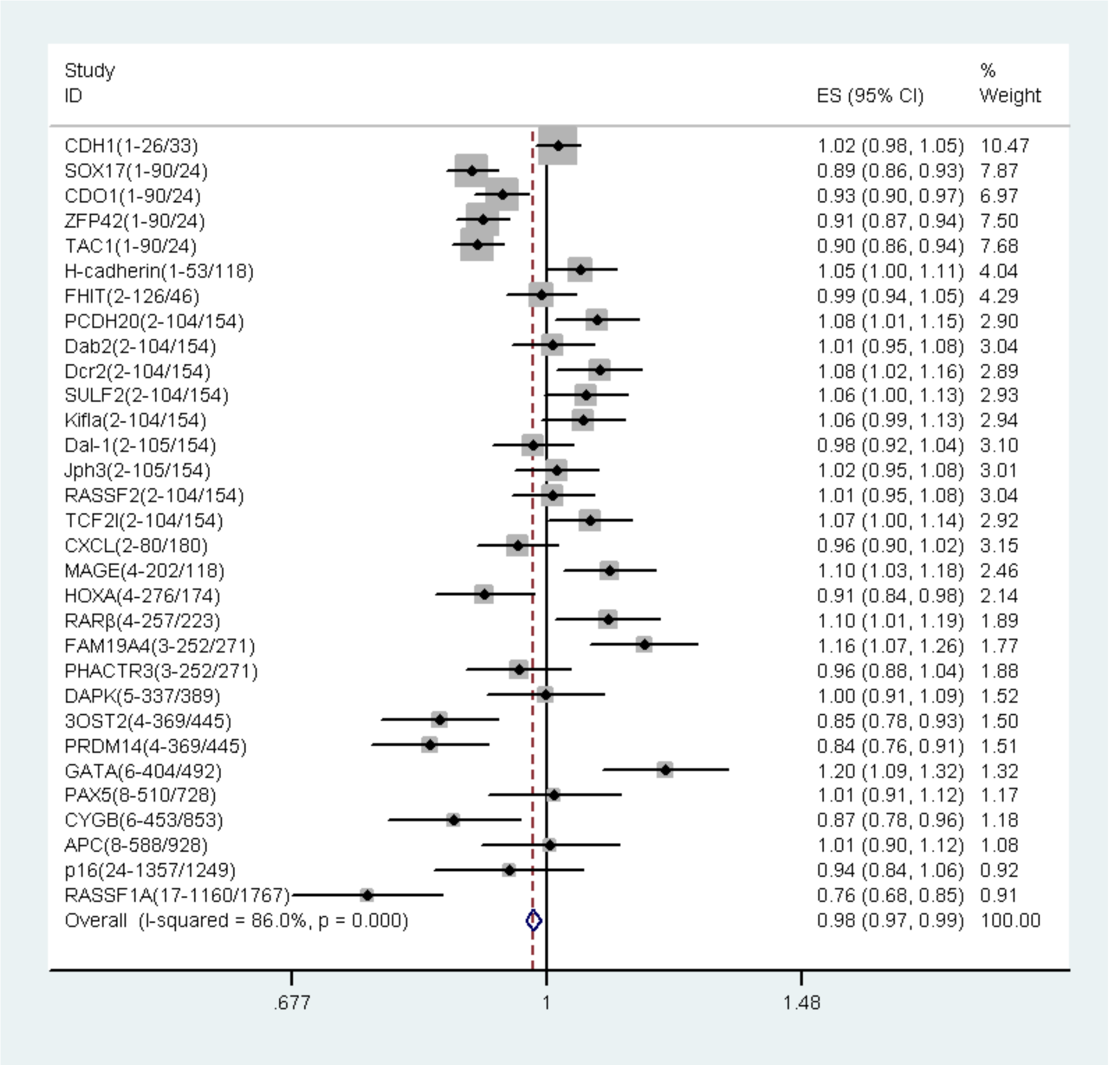

Figure S27. The diagnostic accuracy of 27/*MGMT* compared with the other 31 methylated genes. OR >1 means that 27/*MGMT* had a higher diagnostic accuracy, and 95% CI excluding 1 was considered significant.

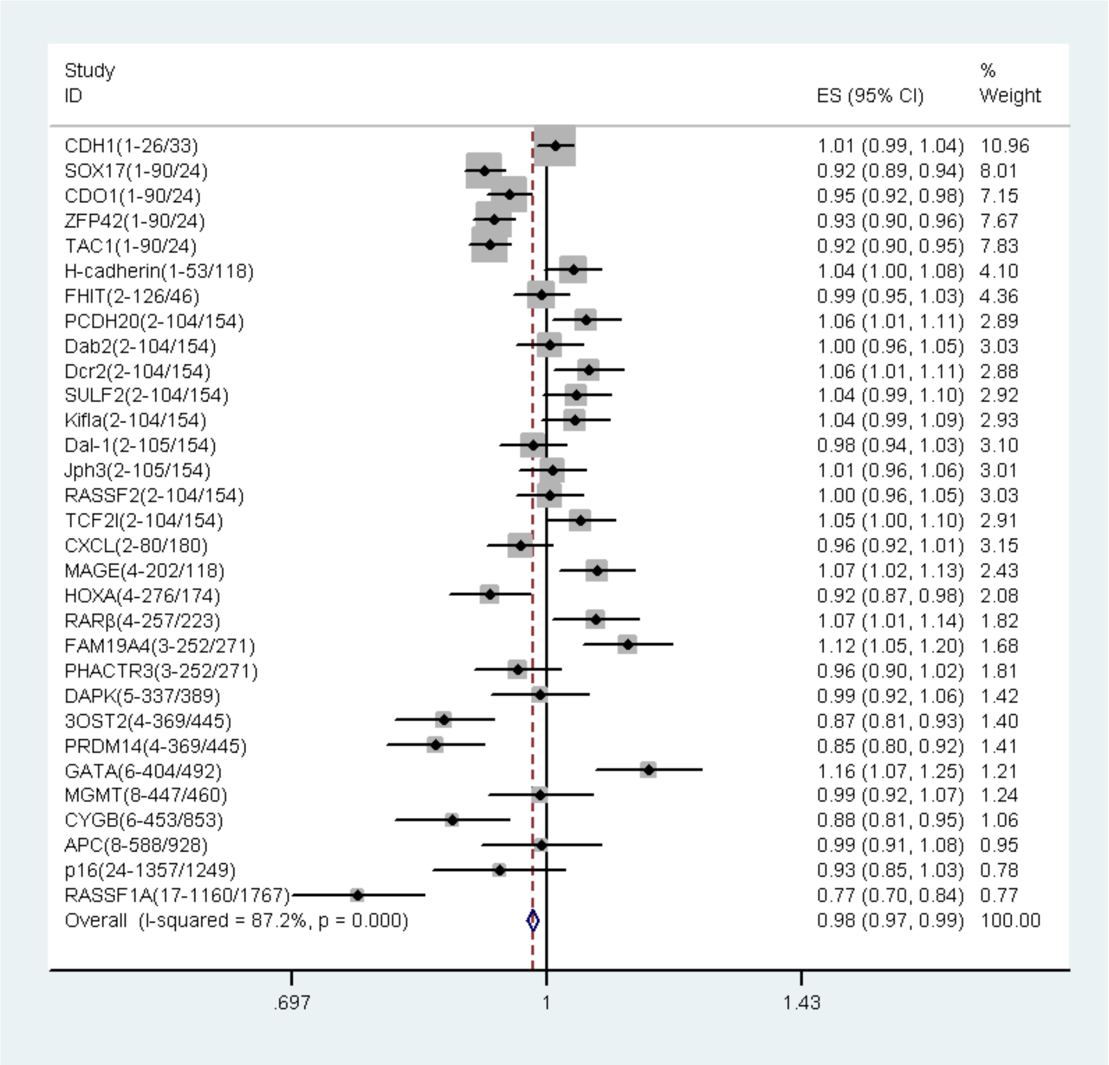

Figure S28. The diagnostic accuracy of 28/*PAX5* compared with the other 31 methylated genes. OR >1 means that 28/*PAX5* had a higher diagnostic accuracy, and 95% CI excluding 1 was considered significant.

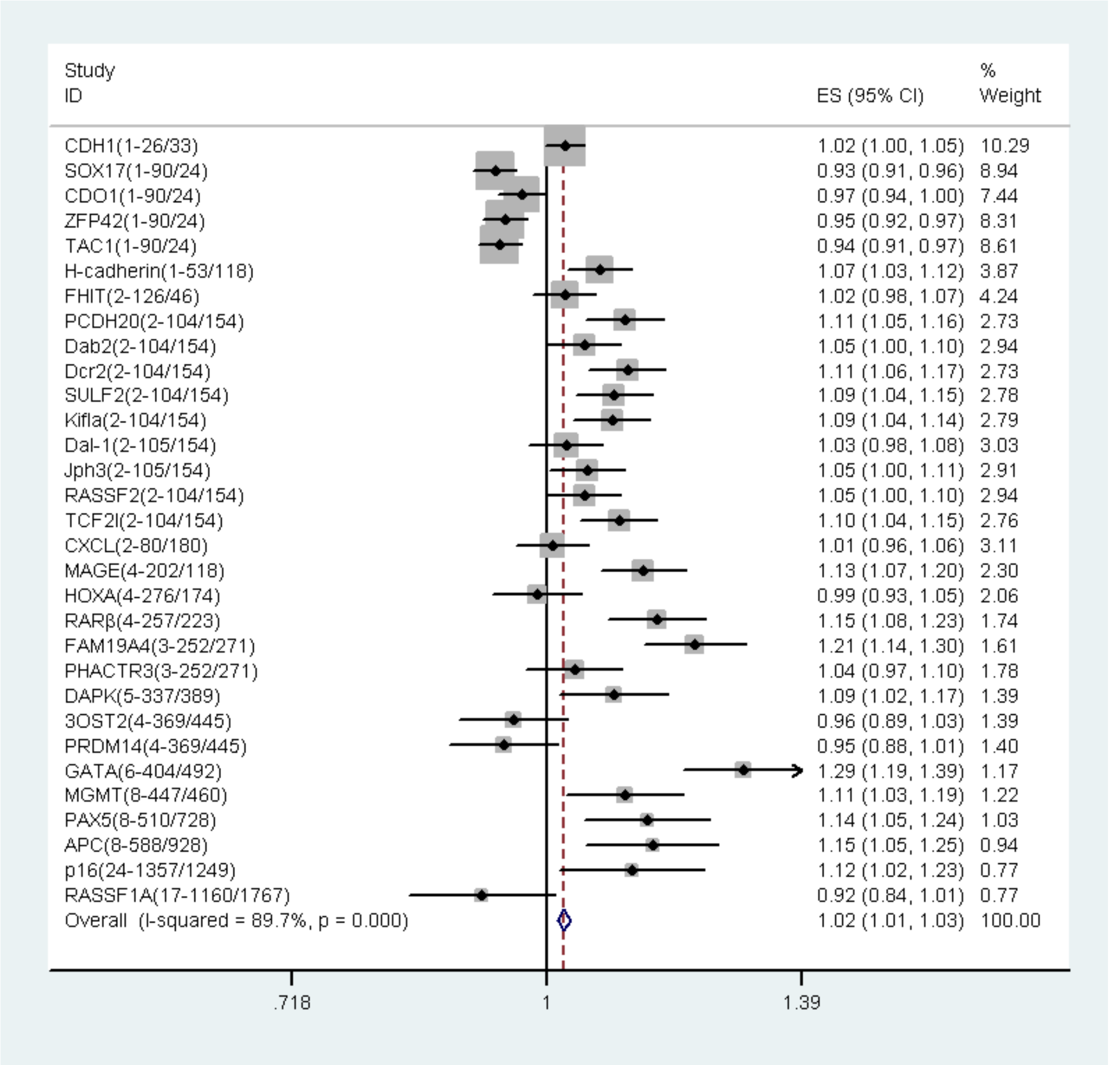

Figure S29. The diagnostic accuracy of 29/*CYGB* compared with the other 31 methylated genes. OR >1 means that 29/*CYGB* had a higher diagnostic accuracy, and 95% CI excluding 1 was considered significant.

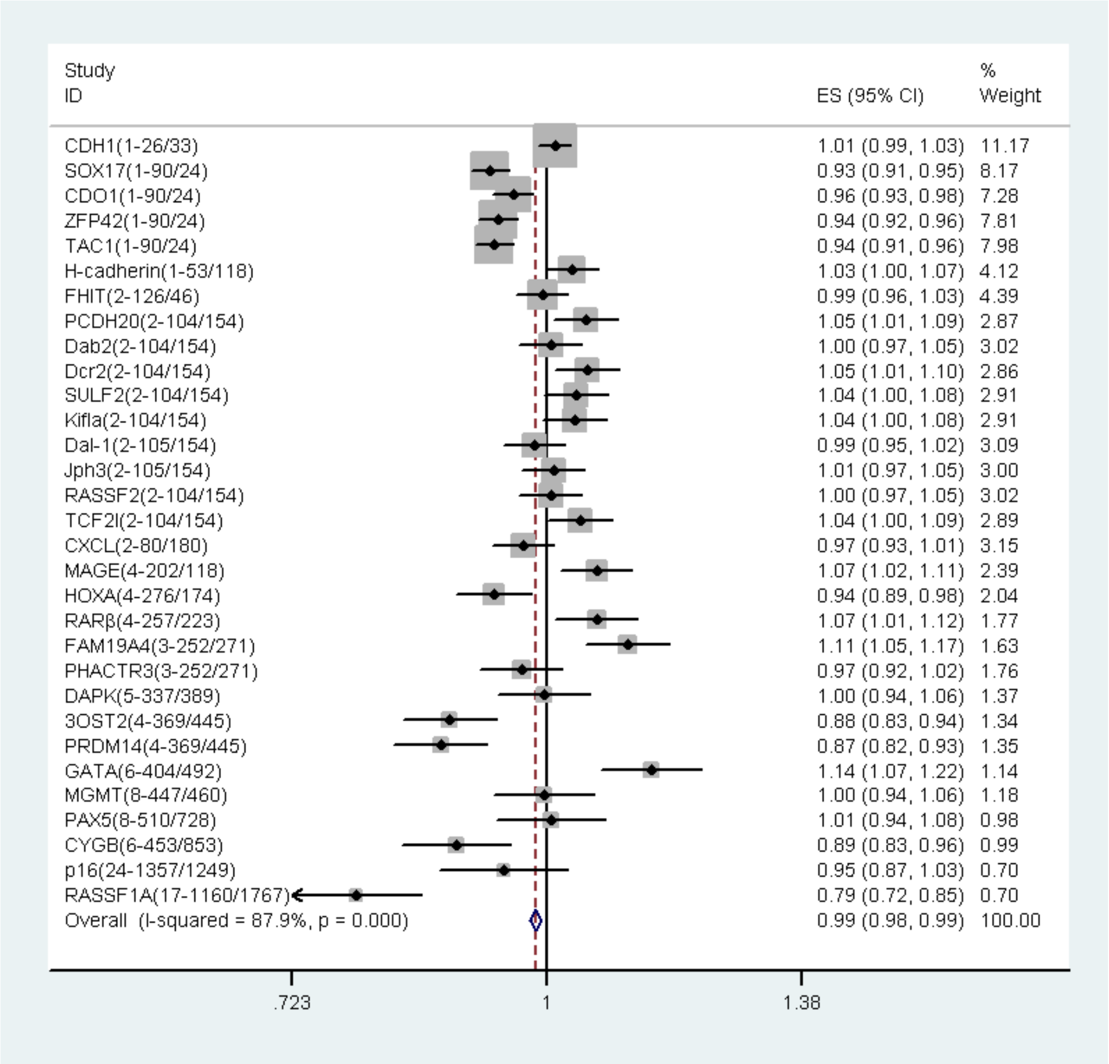

Figure S30. The diagnostic accuracy of 30/*APC* compared with the other 31 methylated genes. OR >1 means that 30/*APC* had a higher diagnostic accuracy, and 95% CI excluding 1 was considered significant.

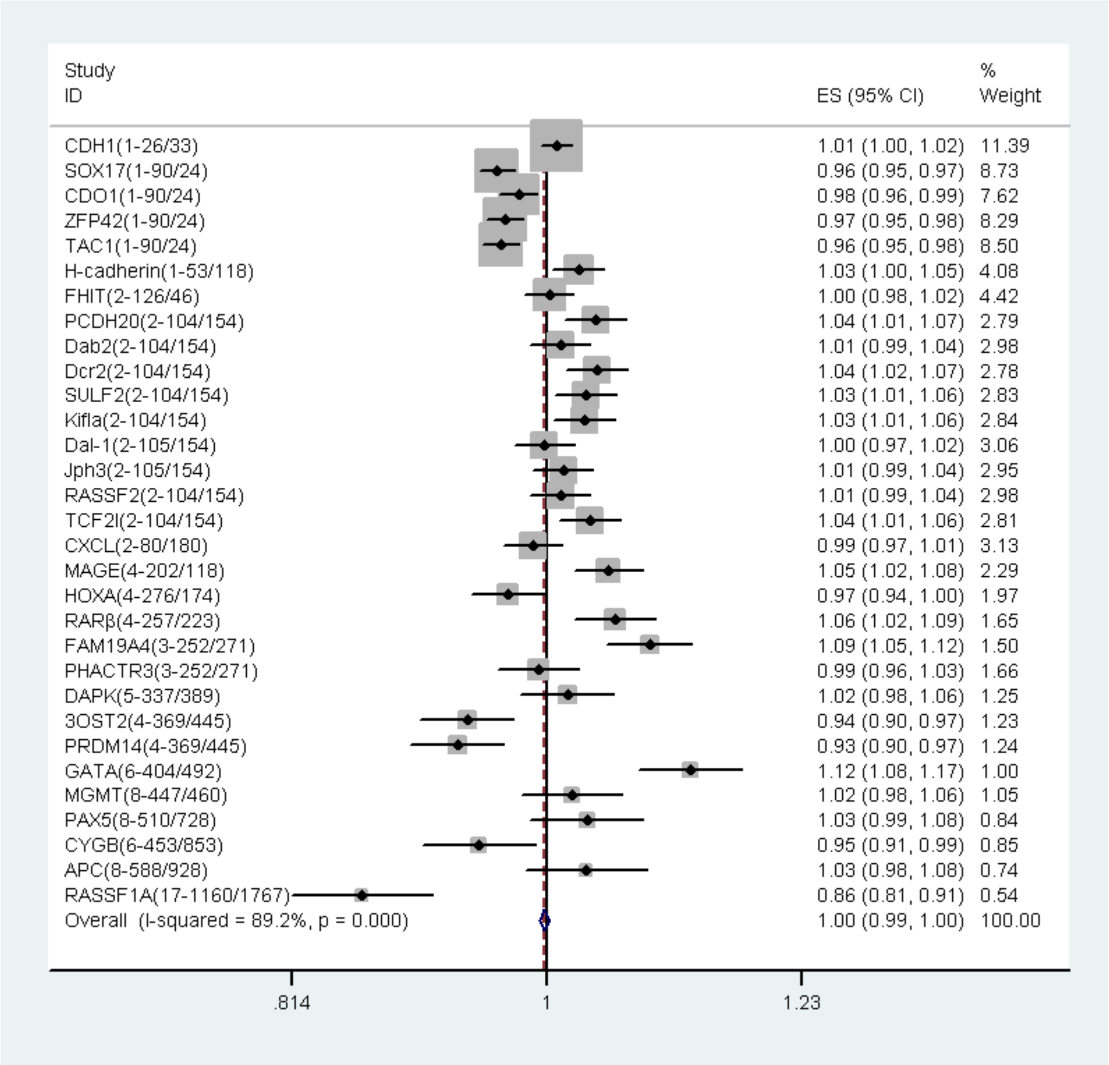

Figure S31. The diagnostic accuracy of 31/*p16* compared with the other 31 methylated genes. OR >1 means that 31/*p16* had a higher diagnostic accuracy, and 95% CI excluding 1 was considered significant.

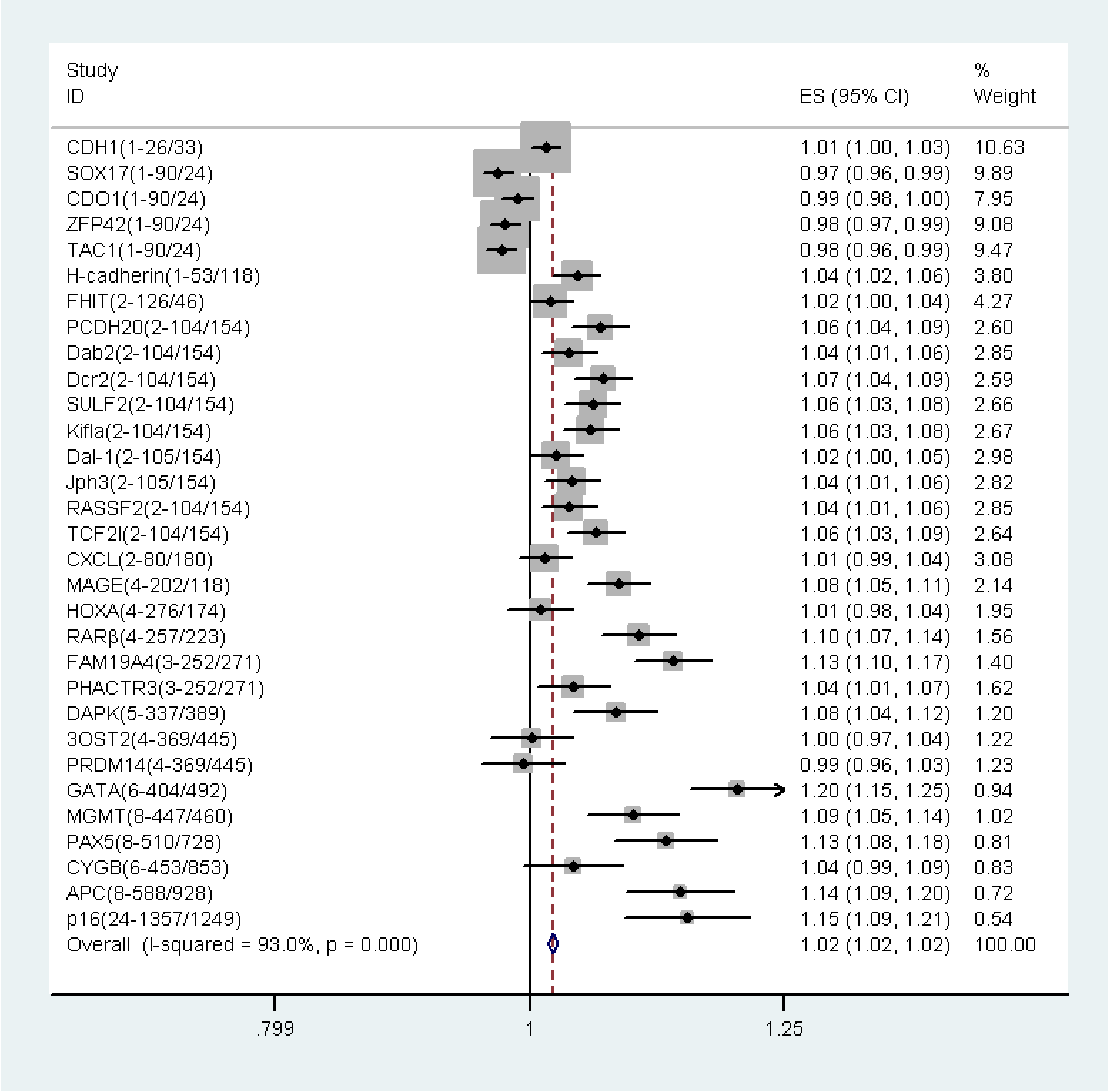

Figure S32. The diagnostic accuracy of 32/*RASSF1A* compared with the other 31 methylated genes. OR >1 means that 32/*RASSF1A* had a higher diagnostic accuracy, and 95% CI excluding 1 was considered significant.

Table S1. Indirect comparisons of 32 genes.

CDH1	4.81(2.33-9.96)	2.59(1.33-5.05)	3.74(1.86-7.51)	4.22(2.07-8.59)	0.38(0.21-0.70)	1.38(0.76-2.56)	0.94(0.53-1.66)	1.27(0.72-2.24)	0.93(0.53-1.63)	1.02(0.58-1.79)	1.03(0.59-1.82)	1.46(0.82-2.58)	1.24(0.70-2.19)	1.26(0.72-2.24)	0.98(0.56-1.74)	1.62(0.91-2.87)	0.92(0.53-1.61)	1.77(1.03-3.07)	1.01(0.59-1.74)	0.89(0.52-1.52)	1.49(0.87-2.57)	1.33(0.78-2.27)	1.89(1.11-3.23)	1.96(1.15-3.34)	0.92(0.54-1.57)	1.32(0.78-2.25)	1.30(0.77-2.19)	1.69(1.00-2.86)	1.31(0.78-2.21)	1.43(0.85-2.41)	1.91(1.14-3.21)
0.21(0.10-0.43)	*SOX17*	0.54(0.28-1.05)	0.78(0.39-1.56)	0.88(0.43-1.79)	0.08(0.04-0.15)	0.29(0.16-0.53)	0.20(0.11-0.35)	0.26(0.15-0.47)	0.19(0.11-0.34)	0.21(0.12-0.37)	0.21(0.12-0.38)	0.30(0.17-0.54)	0.26(0.15-0.46)	0.26(0.15-0.47)	0.20(0.12-0.36)	0.34(0.19-0.60)	0.19(0.11-0.33)	0.37(0.21-0.64)	0.21(0.12-0.36)	0.18(0.11-0.32)	0.31(0.18-0.53)	0.28(0.16-0.47)	0.39(0.23-0.67)	0.41(0.24-0.70)	0.19(0.11-0.33)	0.28(0.16-0.47)	0.27(0.16-0.46)	0.35(0.21-0.60)	0.27(0.16-0.46)	0.30(0.18-0.50)	0.40(0.24-0.67)
0.39(0.20-0.75)	1.86(0.95-3.63)	*CDO1*	1.44(0.76-2.73)	1.63(0.85-3.12)	0.15(0.09-0.25)	0.56(0.32-0.91)	0.36(0.22-0.59)	0.49(0.30-0.80)	0.36(0.22-0.58)	0.39(0.24-0.64)	0.40(0.24-0.65)	0.56(0.34-0.92)	0.48(0.29-0.78)	0.49(0.30-0.80)	0.38(0.23-0.62)	0.63(0.38-1.03)	0.36(0.22-0.57)	0.68(0.43-1.10)	0.39(0.24-0.62)	0.34(0.22-0.54)	0.58(0.36-0.91)	0.51(0.33-0.81)	0.73(0.47-1.15)	0.76(0.48-1.19)	0.36(0.23-0.56)	0.51(0.33-0.80)	0.50(0.32-0.78)	0.65(0.42-1.01)	0.51(0.33-0.79)	0.55(0.36-0.85)	0.74(0.48-1.14)
0.27(0.13-0.54)	1.29(0.64-2.59)	0.69(0.37-1.31)	*ZFP42*	1.13(0.57-2.24)	0.10(0.06-0.18)	0.37(0.21-0.65)	0.25(0.15-0.43)	0.34(0.20-0.58)	0.25(0.15-0.42)	0.27(0.16-0.46)	0.28(0.16-0.47)	0.39(0.23-0.67)	0.33(0.19-0.56)	0.34(0.20-0.58)	0.26(0.16-0.45)	0.43(0.25-0.74)	0.25(0.15-0.42)	0.47(0.28-0.79)	0.27(0.16-0.45)	0.24(0.14-0.39)	0.40(0.24-0.66)	0.36(0.22-0.58)	0.51(0.31-0.83)	0.52(0.32-0.86)	0.25(0.15-0.40)	0.35(0.22-0.58)	0.35(0.21-0.57)	0.45(0.28-0.74)	0.35(0.22-0.57)	0.38(0.24-0.62)	0.51(0.32-0.83)
0.24(0.12-0.48)	1.14(0.56-2.33)	0.61(0.32-1.18)	0.89(0.45-1.75)	*TAC1*	0.09(0.05-0.16)	0.33(0.18-0.590	0.22(0.13-0.39)	0.30(0.17-0.52)	0.22(0.13-0.38)	0.24(0.14-0.42)	0.24(0.14-0.42)	0.35(0.20-0.60)	0.29(0.17-0.51)	0.31(0.17-0.52)	0.23(0.14-0.41)	0.38(0.22-0.67)	0.22(0.13-0.37)	0.42(0.25-0.72)	0.24(0.14-0.41)	0.21(0.13-0.35)	0.35(0.21-0.60)	0.31(0.19-0.53)	0.45(0.27-0.75)	0.46(0.28-0.78)	0.22(0.13-0.36)	0.31(0.19-0.52)	0.31(0.19-0.51)	0.40(0.24-0.66)	0.31(0.19-0.52)	0.34(0.21-0.56)	0.45(0.28-0.75)
2.64(1.44-4.84)	12.70(6.91-23.35)	6.84(4.00-11.68)	9.86(5.56-17.50)	11.13(6.17-20.08)	*H-cadherin*	1.15(0.94-2.23)	2.48(1.65-3.73)	3.35(2.22-5.04)	2.44(1.63-3.67)	2.68(1.79-4.03)	2.73(1.81-4.09)	3.84(2.55-5.80)	3.26(2.17-4.91)	1.32(0.89-1.96)	1.03(0.70-1.51)	4.28(2.83-6.47)	2.43(1.64-3.59)	4.68(3.20-6.85)	2.68(1.85-3.88)	2.34(1.62-3.38)	3.93(2.72-5.70)	3.51(2.45-5.01)	4.99(3.50-7.13)	5.17(3.62-7.39)	2.43(1.72-3.45)	3.49(2.46-4.96)	3.42(2.43-4.83)	4.46(3.16-6.29)	3.47(2.47-4.87)	3.78(2.71-5.28)	5.04(3.61-7.03)
0.72(0.40-1.31)	3.47(1.90-6.14)	1.87(1.10-3.16)	2.69(1.53-4.73)	3.04(1.70-5.43)	0.69(0.45-1.06)	*FHIT*	0.68(0.46-1.01)	0.91(0.62-1.36)	0.67(0.45-0.99)	0.73(0.49-1.09)	0.74(0.50-1.10)	1.05(0.70-1.56)	0.89(0.60-1.32)	0.91(0.62-1.36)	0.71(0.48-1.05)	1.17(0.78-1.740	0.66(0.45-0.97)	1.28(0.89-1.840	0.73(0.51-1.04)	0.64(0.45-0.91)	1.07(0.75-1.53)	0.96(0.68-1.35)	1.36(0.97-1.92)	1.41(1.01-1.990	0.66(0.48-0.93)	0.95(0.68-1.33)	0.93(0.67-1.30)	1.22(0.88-1.69)	0.95(0.68-1.31)	1.03(0.75-1.42)	1.37(1.00-1.89)
1.06(0.60-1.88)	5.12(2.90-9.05)	2.76(1.69-4.50)	3.97(2.33-6.77)	4.49(2.59-7.77)	0.40(0.27-0.61)	1.48(0.99-2.19)	*PCDH20*	1.35(0.95-1.91)	0.98(0.70-1.39)	1.08(0.77-1.53)	1.10(0.78-1.55)	1.55(1.09-2.20)	1.32(0.93-1.86)	1.35(0.95-1.91)	1.05(0.74-1.48)	1.72(1.21-2.46)	0.98(0.71-1.36)	1.89(1.38-2.58)	1.08(0.80-1.46)	0.94(0.70-1.27)	1.59(1.17-2.15)	1.41(1.06-1.88)	2.01(1.51-2.68)	2.08(1.56-2.78)	0.98(0.74-1.29)	1.41(1.07-1.86)	1.38(1.05-1.81)	1.80(1.37-2.35)	1.40(1.07-1.82)	1.52(1.18-1.97)	2.03(1.57-2.62)

0.79(0.45-1.39)	3.79(2.14-6.72)	2.04(1.25-3.35)	2.94(1.72-5.03)	3.32(1.91-5.77)	0.30(0.20-0.45)	1.09(0.74-1.63)	0.74(0.52-1.05)	*Dab2*	0.73(0.51-1.03)	0.80(0.57-1.14)	0.81(0.57-1.15)	1.15(0.80-1.64)	0.97(0.69-1.39)	1.0(0.70-1.42)	0.78(0.55-1.10)	1.28(0.89-1.83)	0.73(0.52-1.01)	1.40(1.02-1.92)	0.80(0.59-1.09)	0.70(0.52-0.95)	1.17(0.86-1.60)	1.05(0.78-1.40)	1.49(1.12-1.99)	1.54(1.15-2.06)	0.73(0.55-0.96)	1.04(0.79-1.38)	1.02(0.78-1.34)	1.33(1.01-1.75)	1.03(0.79-1.35)	1.12(0.87-1.47)	1.50(1.16-1.95)
1.08(0.61-1.91)	5.20(2.94-9.20)	2.80(1.71-4.76)	4.03(2.37-6.88)	4.56(2.63-7.90)	0.41(0.27-0.61)	1.49(1.01-2.22)	1.02(0.72-1.44)	1.37(0.97-1.94)	*Dcr2*	1.10(0.78-1.55)	1.12(0.79-1.58)	1.57(1.11-2.24)	1.34(0.94-1.89)	1.37(0.97-1.94)	1.06(0.75-1.50)	1.75(1.23-2.49)	0.99(0.72-1.38)	1.92(1.40-2.62)	1.10(0.81-1.48)	0.96(0.71-1.29)	1.61(1.19-2.18)	1.43(1.08-1.91)	2.04(1.54-2.72)	2.12(1.59-2.82)	1.00(0.76-1.32)	1.43(1.08-1.89)	1.40(1.07-1.83)	1.82(1.39-2.39)	1.42(1.09-1.85)	1.55(1.20-2.00)	2.06(1.60-2.67)
0.98(0.56-1.74)	4.73(2.68-8.38)	2.55(1.56-4.17)	3.67(2.16-6.26)	4.15(2.39-7.19)	0.37(0.25-0.56)	1.36(0.92-2.03)	0.93(0.65-1.31)	1.25(0.88-1.77)	0.91(0.64-1.29)	*SULF2*	1.02(0.72-1.44)	1.43(1.01-2.04)	1.22(0.86-1.73)	1.25(0.88-1.77)	0.97(0.69-1.37)	1.59(1.12-2.27)	0.91(0.65-1.26)	1.75(1.27-2.39)	1.00(0.74-1.35)	0.87(0.65-1.18)	1.47(1.08-1.99)	1.31(0.98-1.74)	1.86(1.40-2.48)	1.93(1.45-2.57)	0.91(0.69-1.20)	1.30(0.99-1.72)	1.28(0.97-1.67)	1.66(1.27-2.18)	1.29(0.99-1.69)	1.41(1.09-1.82)	1.88(1.45-2.43)
0.97(0.55-1.71)	4.66(2.63-8.25)	2.51(1.53-4.10)	3.62(2.12-6.17)	4.08(2.36-7.08)	0.37(0.24-0.55)	1.34(0.91-1.99)	0.91(0.64-1.29)	1.23(0.87-1.74)	0.90(0.63-1.27)	0.98(0.70-1.39)	*Kifla*	1.41(0.99-2.01)	1.20(0.85-1.70)	1.23(0.87-1.74)	0.95(0.68-1.35)	1.57(1.10-2.24)	0.89(0.64-1.24)	1.72(1.25-2.36)	0.98(0.72-1.33)	0.86(0.64-1.16)	1.44(1.07-1.96)	1.29(0.97-1.71)	1.83(1.38-2.44)	1.90(1.42-2.53)	0.89(0.68-1.18)	1.28(0.97-1.70)	1.26(0.96-1.65)	1.64(1.25-2.15)	1.27(0.97-1.66)	1.39(1.07-1.80)	1.85(1.43-2.39)
0.69(0.39-1.22)	3.31(1.86-5.87)	1.78(1.08-2.92)	2.57(1.50-4.39)	2.90(1.67-5.04)	0.26(0.17-0.39)	0.95(0.64-1.42)	0.65(0.45-0.92)	0.87(0.61-1.24)	0.64(0.45-0.90)	0.70(0.49-0.99)	0.71(0.50-1.01)	*Dal-1*	0.85(0.60-1.21)	0.87(0.61-1.24)	0.68(0.48-0.96)	1.11(0.78-1.60)	0.63(0.45-0.88)	1.22(0.88-1.68)	0.70(0.51-0.95)	0.61(0.45-0.83)	1.02(0.75-1.40)	0.91(0.68-1.22)	1.30(0.97-1.74)	1.35(1.00-1.81)	0.63(0.48-0.84)	0.91(0.68-1.21)	0.89(0.68-1.18)	1.16(0.88-1.53)	0.90(0.69-1.19)	0.98(0.76-1.28)	1.31(1.01-1.71)
0.81(0.46-1.43)	3.89(2.20-6.89)	2.09(1.28-3.43)	3.02(1.77-5.15)	3.41(1.96-5.92)	0.31(0.20-0.46)	1.12(0.76-1.67)	0.76(0.54-1.08)	1.03(0.72-1.46)	0.75(0.53-1.06)	0.82(0.58-1.17)	0.83(0.59-1.18)	1.18(0.83-1.68)	*Jph3*	1.03(0.72-1.46)	0.80(0.56-1.13)	1.31(0.92-1.87)	0.74(0.53-1.04)	1.43(1.04-1.97)	0.82(0.60-1.11)	0.72(0.53-0.97)	1.20(0.89-1.64)	1.07(0.80-1.43)	1.53(1.15-2.04)	1.58(1.19-2.12)	0.75(0.56-0.99)	1.07(0.81-1.42)	1.05(0.80-1.38)	1.37(0.04-1.80)	1.06(0.81-1.39)	1.16(0.90-1.50)	1.54(1.19-2.00)
0.79(0.45-1.39)	3.79(2.14-6.72)	2.04(1.25-3.35)	2.94(1.72-5.03)	3.32(1.19-5.77)	0.76(0.51-1.12)	1.09(0.74-1.27)	0.74(0.52-1.05)	1.00(0.70-1.42)	0.73(0.51-1.03)	0.80(0.57-1.14)	0.81(0.57-1.15)	1.15(0.80-1.64)	0.97(0.69-1.39)	*RASSF2*	0.78(0.55-1.10)	1.28(0.89-1.83)	0.72(0.52-1.01)	1.40(1.02-1.92)	0.80(0.59-1.09)	0.70(0.52-0.95)	1.17(0.86-1.60)	1.04(0.78-1.40)	1.49(1.12-1.99)	1.54(1.15-2.06)	0.73(0.55-0.96)	1.04(0.79-1.38)	1.02(0.78-1.34)	1.33(1.01-1.75)	1.03(0.79-1.35)	1.13(0.87-1.47)	1.50(1.16-195)
1.01(0.58-1.79)	4.88(2.76-8.64)	2.63(1.61-4.30)	3.79(2.22-6.46)	4.28(2.47-7.42)	0.97(0.66-1.44)	1.41(0.95-2.09)	0.95(0.68-1.35)	1.29(0.91-1.83)	0.94(0.67-1.33)	1.03(0.73-1.46)	1.05(0.74-1.48)	1.48(1.04-2.10)	1.26(0.89-1.78)	1.29(0.91-1.83)	*TCF21*	1.64(1.15-2.34)	0.93(0.67-1.30)	1.80(1.32-2.47)	1.03(0.76-1.39)	0.90(0.68-1.21)	1.51(1.12-2.05)	1.35(1.01-1.80)	1.92(1.44-2.56)	1.99(1.49-2.65)	0.94(0.71-1.24)	1.34(1.02-1.78)	1.32(1.01-1.72)	1.71(1.31-2.45)	1.33(1.02-1.74)	1.45(1.12-1.88)	1.94(1.50-2.51)
0.62(	2.97(	1.60(	2.30(	2.60(	0.23(	0.85(	0.58(	0.78(	0.57(	0.63(	0.64(	0.90(	0.76(	0.78(	0.61(		0.57(	1.09(	0.63(	0.55(	0.92(	0.82(	1.17(	1.21(	0.57(	0.82(	0.80(	1.04(	0.81(	0.88(	1.18(

0.35-1.09)	1.67-5.28)	0.97-2.63)	1.34-3.95)	1.49-4.53)	0.16-0.35)	0.57-1.28)	0.41-0.83)	0.55-1.12)	0.40-0.81)	0.44-0.89)	0.45-0.91)	0.63-1.29)	0.53-1.09)	0.55-1.12)	0.43-0.87)	*CXCL*	0.41-0.80)	0.79-1.51)	0.46-0.86)	0.40-0.75)	0.67-1.26)	0.61-1.10)	0.87-1.57)	0.90-1.63)	0.43-0.76)	0.61-1.09)	0.60-1.06)	0.79-1.38)	0.62-1.07)	0.68-1.16)	0.90-1.54)
1.09(0.62-1.90)	5.23(2.99-9.15)	2.81(1.74-4.55)	4.06(2.41-6.84)	4.58(2.67-7.85)	0.41(0.28-0.61)	1.51(1.03-2.20)	1.02(0.74-1.42)	1.38(0.99-1.92)	1.01(0.72-1.40)	1.10(0.80-1.53)	1.12(0.81-1.56)	1.58(1.13-2.21)	1.34(0.97-1.87)	1.38(0.99-1.92)	1.07(0.77-1.49)	1.76(1.26-2.47)	*MAGE*	1.93(1.43-2.59)	1.10(0.83-1.46)	0.96(0.73-1.27)	1.62(1.22-2.15)	1.44(1.11-1.88)	2.05(1.58-2.68)	2.13(1.63-2.78)	1.00(0.78-1.29)	1.44(1.11-1.86)	1.41(1.10-1.80)	1.83(1.43-2.35)	1.43(1.12-1.82)	1.56(1.23-1.96)	2.07(1.64-2.62)
0.56(0.33-0.98)	2.71(1.56-4.71)	1.46(0.91-2.34)	2.11(1.26-3.52)	2.38(1.40-4.04)	0.21(0.15-0.31)	0.78(0.54-1.30)	0.53(0.39-0.73)	0.72(0.52-0.98)	0.52(0.38-0.71)	0.57(0.42-0.79)	0.58(0.43-0.80)	0.82(0.60-1.13)	0.70(0.51-0.96)	0.72(0.52-0.98)	0.56(0.41-0.76)	0.91(0.66-1.26)	0.52(0.39-0.70)	*HOXA*	0.57(0.44-0.75)	0.50(0.39-0.65)	0.84(0.64-1.10)	0.75(0.58-0.96)	1.07(0.83-1.37)	1.10(0.86-1.42)	0.52(0.41-0.66)	0.75(0.59-0.95)	0.73(0.58-0.92)	0.95(0.76-1.20)	0.74(0.59-0.93)	0.81(0.65-1.00)	1.08(0.87-1.33)
0.99(0.57-1.70)	4.75(2.75-8.20)	2.56(1.61-4.06)	3.68(2.22-6.11)	4.16(2.46-7.03)	0.37(0.26-0.54)	1.37(0.96-1.96)	0.93(0.69-1.26)	1.25(0.92-1.70)	0.91(0.67-1.24)	1.00(0.74-1.36)	1.02(0.75-1.38)	1.44(1.05-1.96)	1.22(0.90-1.66)	1.25(0.92-1.70)	0.97(0.72-1.32)	1.60(1.17-2.19)	0.91(0.68-1.21)	1.75(1.34-2.29)	*RARβ*	0.87(0.68-1.12)	1.47(1.14-1.89)	1.31(1.04-1.66)	1.87(1.48-2.36)	1.93(1.53-2.44)	0.91(0.73-1.14)	1.31(1.04-1.63)	1.28(1.03-1.58)	1.67(1.35-2.06)	1.30(1.05-1.59)	1.41(1.16-1.72)	1.88(1.55-2.29)
1.13(0.66-1.94)	5.43(3.15-9.35)	2.92(1.85-4.63)	4.21(2.55-6.97)	4.76(2.82-8.02)	0.43(0.30-0.62)	1.57(1.10-2.23)	1.06(0.79-1.43)	1.43(0.06-1.94)	1.04(0.78-1.41)	1.15(0.85-1.55)	1.16(0.86-1.57)	1.64(1.21-2.23)	1.40(1.03-1.89)	1.43(1.06-1.94)	1.11(0.82-1.50)	1.83(1.34-2.49)	1.04(0.79-1.37)	2.00(1.54-2.60)	1.14(0.89-1.47)	*FAM19A4*	1.68(1.31-2.15)	1.50(1.20-1.88)	2.13(1.70-2.68)	2.21(1.76-2.76)	1.04(0.84-1.29)	1.49(1.20-1.86)	1.46(1.19-1.80)	1.91(1.55-2.34)	1.48(1.21-1.81)	1.62(1.34-1.95)	2.15(1.78-2.60)
0.67(0.39-1.15)	3.23(1.87-5.57)	1.74(1.10-2.76)	2.51(1.51-4.16)	2.83(1.68-4.78)	0.25(0.18-0.37)	0.93(0.65-1.33)	0.63(0.47-0.85)	0.85(0.63-1.16)	0.62(0.46-0.84)	0.68(0.50-0.92)	0.69(0.51-0.94)	0.98(0.72-1.33)	0.83(0.61-1.13)	0.85(0.63-1.16)	0.66(0.49-0.90)	1.09(0.80-1.49)	0.62(0.47-0.82)	1.19(0.91-1.55)	0.68(0.53-0.88)	0.59(0.46-0.76)	*PHACTR3*	0.89(0.71-1.12)	1.27(1.01-1.60)	1.31(1.04-1.66)	0.62(0.50-0.77)	0.89(0.71-1.11)	0.87(0.70-1.08)	1.13(0.92-1.40)	0.88(0.72-1.08)	0.96(0.79-1.17)	1.28(1.05-1.56)
0.75(0.44-1.28)	3.62(2.12-6.20)	1.95(1.24-3.06)	2.81(1.71-4.62)	2.13(1.26-3.58)	0.29(0.20-0.41)	1.04(0.74-1.47)	0.47(0.35-0.64)	0.96(0.72-1.28)	0.70(0.52-0.93)	0.77(0.57-1.02)	0.78(0.58-1.04)	1.10(0.82-1.47)	0.93(0.70-1.24)	0.95(0.72-1.28)	0.74(0.56-0.99)	1.22(0.91-1.64)	0.69(0.53-0.90)	1.34(1.04-1.71)	0.76(0.60-0.96)	0.67(0.53-0.84)	1.12(0.89-1.42)	*DAPK*	1.42(1.15-1.76)	1.48(1.19-1.82)	0.69(0.57-0.85)	1.00(0.82-1.22)	0.98(0.81-1.18)	1.27(1.05-1.54)	0.99(0.83-1.19)	1.08(0.91-1.28)	1.44(1.21-1.70)
0.53(0.31-0.90)	2.54(1.49-4.35)	1.37(0.87-2.15)	1.97(1.20-3.24)	2.23(1.33-3.73)	0.20(0.14-0.29)	0.73(0.52-1.03)	0.50(0.37-0.66)	0.67(0.50-0.90)	0.49(0.37-0.65)	0.54(0.40-0.72)	0.55(0.41-0.73)	0.77(0.57-1.03)	0.65(0.49-0.87)	0.67(0.50-0.90)	0.52(0.39-0.69)	0.86(0.64-1.15)	0.49(0.37-0.63)	0.94(0.73-1.20)	0.54(0.42-0.68)	0.47(0.37-0.59)	0.79(0.63-0.99)	0.70(0.57-0.87)	*3OST2*	1.04(0.84-1.28)	0.49(0.40-0.59)	0.70(0.57-0.85)	0.69(0.57-0.83)	0.89(0.74-1.08)	0.69(0.58-0.83)	0.76(0.64-0.90)	1.01(0.85-1.19)
0.51(0.30-0.87)	2.46(1.44-4.20)	1.32(0.84-2.08)	1.91(1.16-3.13)	2.15(1.29-3.60)	0.19(0.14-0.28)	0.71(0.50-0.99)	0.48(0.36-0.64)	0.65(0.49-0.87)	0.47(0.36-0.63)	0.52(0.39-0.69)	0.53(0.40-0.70)	0.74(0.55-1.00)	0.63(0.47-0.84)	0.65(0.49-0.87)	0.50(0.38-0.67)	0.83(0.62-1.11)	0.47(0.36-0.61)	0.91(0.71-1.16)	0.52(0.41-0.65)	0.45(0.36-0.57)	0.76(0.60-0.96)	0.68(0.55-0.84)	0.70(0.59-0.82)	*PRDM14*	0.47(0.39-0.58)	0.68(0.55-0.83)	0.66(0.55-0.80)	0.86(0.71-1.04)	0.67(0.56-0.80)	0.73(0.62-0.87)	0.97(0.82-1.15)

1.08(0.64-1.84)	5.22(3.07-8.88)	2.81(1.80-4.39)	4.05(2.48-6.62)	4.57(2.75-7.61)	0.41(0.29-0.58)	1.51(1.08-2.10)	1.02(0.77-1.35)	1.38(1.04-1.82)	1.00(0.76-1.32)	1.10(0.84-1.46)	1.12(0.85-1.48)	1.58(1.19-2.10)	1.34(1.01-1.78)	1.38(1.04-1.82)	1.07(0.81-1.41)	1.76(1.32-2.34)	1.00(0.77-1.29)	1.92(1.52-2.44)	1.10(0.88-1.37)	0.96(0.78-1.19)	1.62(1.30-2.02)	1.44(1.18-1.76)	2.05(1.68-2.50)	2.12(1.74-2.59)	*GATA*	1.43(1.19-1.73)	1.41(1.18-1.67)	1.83(1.54-2.18)	1.42(1.21-1.68)	1.55(1.33-1.81)	2.07(1.78-2.41)
0.76(0.45-1.28)	3.63(2.14-6.19)	1.96(1.25-3.06)	2.82(1.73-4.62)	3.19(1.91-5.31)	0.29(0.20-0.41)	1.04(0.75-1.49)	0.71(0.54-0.94)	0.96(0.72-1.27)	0.70(0.53-0.92)	0.77(0.58-1.01)	0.78(0.59-1.03)	1.10(0.83-1.46)	0.93(0.71-1.24)	0.96(0.72-1.27)	1.07(0.81-1.41)	1.22(0.92-1.63)	0.70(0.54-0.90)	1.34(1.06-1.70)	0.77(0.61-0.96)	0.67(0.54-0.83)	1.13(0.90-1.41)	1.00(0.82-1.23)	1.43(1.17-1.75)	1.48(1.21-1.81)	0.70(0.58-0.84)	*MGMT*	0.98(0.82-1.17)	1.28(1.07-1.52)	0.99(0.84-1.17)	1.08(0.93-1.26)	1.44(1.24-1.68)
0.77(0.46-1.30)	3.71(2.19-6.29)	2.00(1.29-3.11)	2.88(1.77-4.69)	3.25(1.96-5.39)	0.29(0.21-0.41)	1.07(0.77-1.49)	0.73(0.55-0.95)	0.98(0.74-1.29)	0.71(0.55-0.94)	0.78(0.60-1.03)	0.90(0.61-1.04)	1.12(0.85-1.48)	0.95(0.73-1.25)	0.97(0.74-1.23)	0.76(0.58-0.99)	1.25(0.94-1.66)	0.71(0.56-0.91)	1.37(1.09-1.72)	0.78(0.63-0.97)	0.68(0.56-0.84)	1.15(0.93-1.42)	1.02(0.85-1.24)	1.46(1.21-1.76)	1.51(1.25-1.82)	0.71(0.60-0.85)	1.02(0.86-1.22)	*PAX5*	1.30(1.11-1.53)	1.01(0.87-1.18)	1.10(0.96-1.27)	1.47(1.28-1.69)
0.59(0.35-1.00)	2.85(1.68-4.83)	1.53(0.99-2.39)	2.21(1.36-3.60)	2.50(1.51-4.14)	0.22(0.16-0.32)	0.82(0.59-1.14)	0.56(0.43-0.73)	0.75(0.57-0.99)	0.55(0.42-0.72)	0.60(0.46-0.79)	0.61(0.47-0.80)	0.86(0.65-1.14)	0.73(0.56-0.96)	0.75(0.57-0.99)	0.58(0.45-0.77)	0.96(0.73-1.27)	0.55(0.43-0.70)	1.05(0.84-1.32)	0.60(0.49-0.74)	0.52(0.43-0.65)	0.88(0.71-1.09)	0.79(0.65-0.95)	1.12(0.93-1.35)	1.16(0.96-1.40)	0.55(0.46-0.65)	0.78(0.66-0.94)	0.77(0.65-0.90)	*CYGB*	0.78(0.67-0.91)	0.85(0.74-0.98)	1.13(0.98-1.30)
0.76(0.45-1.28)	3.67(2.17-6.20)	1.97(1.27-3.06)	2.85(1.75-4.62)	3.21(1.94-5.31)	0.29(0.21-0.41)	1.06(0.76-1.46)	0.72(0.55-0.93)	0.97(0.74-1.27)	0.71(0.54-0.92)	0.77(0.59-1.01)	0.79(0.60-1.03)	1.11(0.84-1.46)	0.94(0.72-1.23)	0.97(0.74-1.27)	0.75(0.58-0.98)	1.23(0.94-1.63)	0.70(0.55-0.89)	1.35(1.08-1.69)	0.77(0.63-0.95)	0.68(0.55-0.83)	1.13(0.92-1.40)	1.01(0.84-1.21)	1.44(1.20-1.73)	1.49(1.24-1.79)	0.70(0.60-0.83)	1.01(0.85-1.19)	0.99(0.85-1.15)	1.29(1.10-1.50)	*APC*	1.09(0.96-1.24)	1.45(1.28-1.66)
0.70(0.42-1.17)	3.36(1.99-5.66)	1.81(1.17-2.79)	2.61(1.61-4.22)	2.95(1.79-4.85)	0.26(0.19-0.37)	0.96(0.71-1.33)	0.66(0.51-0.85)	0.89(0.68-1.15)	0.65(0.50-0.84)	0.71(0.55-0.92)	0.72(0.56-0.93)	1.02(0.78-	0.86(0.67-1.12)	0.89(0.68-1.15)	0.69(0.53-0.89)	1.13(0.87-1.48)	0.64(0.51-0.81)	1.24(1.00-1.53)	0.71(0.58-0.86)	0.62(0.51-0.75)	1.04(0.86-1.27)	0.93(0.78-1.10)	1.32(1.12-1.56)	1.37(1.16-1.62)	0.64(0.55-0.75)	0.92(0.79-1.08)	0.91(0.79-1.04)	1.18(1.03-1.36)	0.92(0.81-1.04)	*P16*	1.33(1.19-1.49)
0.52(0.31-0.88)	2.52(1.50-4.25)	1.36(0.88-2.09)	1.96(1.21-3.16)	2.21(1.34-3.64)	0.20(0.14-0.28)	0.73(0.53-1.0)	0.49(0.38-0.64)	0.66(0.51-0.86)	0.48(0.38-0.63)	0.53(0.41-0.69)	0.54(0.42-0.70)	0.76(0.59-0.99)	0.65(0.50-0.84)	0.66(0.51-0.86)	0.52(0.40-0.67)	0.85(0.65-1.11)	0.48(0.38-0.61)	0.93(0.75-1.15)	0.53(0.44-0.65)	0.46(0.39-0.56)	0.78(0.64-0.95)	0.70(0.59-0.82)	0.99(0.84-1.17)	1.03(0.87-1.22)	0.48(0.42-0.56)	0.69(0.59-0.81)	0.68(0.59-0.78)	0.88(0.77-1.02)	0.69(0.60-0.78)	0.75(0.67-0.84)	*RASS F1A*

In column the methylated gene compared with the other 31 methylated genes; in row the other 31 methylated genes compared with the methylated gene. OR >1 means that the methylated gene had a higher diagnostic accuracy, and 95% CI excluding 1 was considered significant.
